# The unique chemical and microbiological signatures of an array of bottled drinking water

**DOI:** 10.3389/fmicb.2024.1441142

**Published:** 2024-09-16

**Authors:** Yasmeen M. Nadreen, Johannes S. Vrouwenvelder, Pascal E. Saikaly, Graciela Gonzalez-Gil

**Affiliations:** ^1^Water Desalination and Reuse Center, King Abdullah University of Science and Technology, Thuwal, Saudi Arabia; ^2^Environmental Science and Engineering Program, Biological and Environmental Science and Engineering Division, King Abdullah University of Science and Technology, Thuwal, Saudi Arabia

**Keywords:** microbiological stability, microbial growth potential, plastic versus glass, reverse osmosis, environmental sustainability

## Abstract

The bottled drinking water market has seen significant growth and diversification, yet the selection criteria lack scientific basis, as all must adhere to stringent health standards. Prior studies predominantly focused on chemical quality, with limited assessments of microbial quality using methods prone to underestimation. Moreover, insufficient research explores the impact of packaging materials and temperatures optimal for mesophilic growth on microbial quality. To understand the unique characteristics and justify the distinction among different types of bottled waters, a comprehensive analysis encompassing both chemical and microbiological aspects is imperative. Addressing these gaps, our study examines 19 diverse bottled water brands comprising purified, mineral, artesian, and sparkling water types from Saudi Arabia and abroad. Our findings reveal distinct chemical compositions among bottled waters, with notable variations across types. Flow cytometry analysis reveals significant differences in bacterial content among water types, with natural mineral waters having the highest concentrations and treated purified waters the lowest. Bacterial content in plastic-bottled mineral water suggests it may be higher than in glass-bottled water. Flow cytometry fingerprints highlight separate microbial communities for purified and mineral waters. Additionally, temperatures favorable for mesophilic growth reveal varying microbial responses among different types of bottled waters. Some variation is also observed in mineral water bottled in plastic versus glass, suggesting potential differences that warrant further investigation. 16S rRNA gene sequencing identifies unique microbial taxa among different mineral waters. Overall, our study underscores that all bottled waters meet health regulations. Furthermore, the combined chemical and microbial profiles may serve as authenticity indicators for distinct bottled water types. This study can serve as a basis for future research on the environmental impact of bottled water transportation, suggesting that locally produced water may offer a more sustainable option.

## 1 Introduction

The demand for bottled drinking water has surged globally, driven by diverse brands marketing water from various sources (Tarver, 2008; Rani et al., 2012). Consumer preferences are influenced by factors such as taste, brand advertising, and perceived quality based on source or mineral content (Doria, 2006; Ferrier, 2001; Studlick and Bain, 1980). However, there is a lack of scientific evidence supporting the health benefits of one water type over another, as all must adhere to stringent health standards (Bullers, 2002; Tarver, 2008). Environmental considerations also arise, depending on water origin and transportation methods (Gleick and Cooley, 2009; Hassan, 2016; Rooy, 2018). To justify the distinctions among bottled waters, a comprehensive analysis encompassing physical, chemical, and microbiological features is necessary to discern and compare the quality and intrinsic features of different types of bottled water. Studies investigating the chemical and microbiological quality of bottled waters tend to focus on local brands and their potential pathogenicity, rather than providing a comprehensive characterization across a diverse range of bottled water types (Cohen et al., 2022b; Ghanbarian et al., 2022; Cohen et al., 2022a).

Chemical compositions of distinct bottled waters vary (Ferrier, 2001; Bitton, 2014). Studies have scrutinized mineral concentrations to verify label values and compliance with regulations (Abouleish, 2016; Mahajan et al., 2006; Azlan et al., 2012; Alfadul and Khan, 2011; Ahmad and Bajahlan, 2009; Maddah and Alzhrani, 2017; Alabdula’aly and Khan, 1999). However, research primarily focused on water quality and compliance, rather than exploring variations among bottled water types. Limited studies have used multivariate recognition techniques to categorize local bottled waters but neglected microbiological properties (Güler, 2007).

Bottled water microbiology differs widely based on its source and purification methods, with mineral waters maintaining their natural microbial flora while purified waters undergo treatment and disinfection processes (Bitton, 2014). Previous studies investigating microbiological properties of mineral waters (Ahmad and Bajahlan, 2009; Varga, 2011; Daood, 2008; Zamberlan da Silva et al., 2008) and purified bottled waters (Alotaibi and Eed, 2009) predominantly relied on heterotrophic plate count (HPC), often underestimating microbial numbers compared to advanced methods like flow cytometry (FCM) (Van Nevel et al., 2017). While FCM has been utilized in studying drinking water treatments and quality, relevant investigations into microbial concentrations across different bottled water types remain scarce (Cheswick et al., 2019; Hammes et al., 2010; Hammes et al., 2008; Sousi et al., 2020; Prest et al., 2016a). Employing FCM can provide more accurate microbial assessments and reveal potential differences between bottled water types.

Bottled water attributes, including the choice of plastic or glass packaging, can significantly affect microbial content. Plastic containers often harbor more microbes than glass ones (Bitton, 2014; Bischofberger et al., 1990), although their precise impact on microbial growth remains unclear. Environmental factors such as elevated temperatures can further influence the microbial quality of bottled waters (Bischofberger et al., 1990; Raj, 2005). Given the diversity of bottled water types and sources, the impact of high local temperatures over time on microbial growth in waters with varying microbial content remains unexplored. Utilizing sensors, such as with FCM, in an online setting provides a promising avenue to monitor changes in microbial quality over time, particularly in response to external factors like temperature (Hammes and Besmer, 2018).

Additionally, comprehensive studies on microbial community compositions in diverse bottled waters are lacking. For instance, one study on bottled water microbial communities excluded natural mineral waters (Brumfield et al., 2020), while others focused solely on local bottled mineral waters (Carraturo et al., 2021; Falcone-Dias et al., 2015). Despite some research exploring microbial flora in mineral water, these studies often emphasize pathogenic species, potentially overlooking valuable insights into water sources (Bischofberger et al., 1990; Hunter, 1993). Notably, a study on mineral bottled waters revealed diverse microbial communities unique to each sample, highlighting the need for further exploration (Sala-Comorera et al., 2020). Concurrently collecting microbial and chemical composition data will be crucial for safeguarding the authenticity of natural mineral waters facing challenges from water stress in diverse locations.

This study aims to compare the chemical and microbiological quality of four types of bottled water in Saudi Arabia and imported from other countries. Utilizing advanced analytical techniques, including ion chromatography, spectroscopy, FCM, and 16S rRNA gene sequencing, we explore unique compositions and microbial communities in purified, mineral, artesian, and sparkling bottled waters. Moreover, we investigate the different types of water and bottling materials under conditions optimized for mesophilic growth to assess microbial concentrations and growth potential. We aim to reveal the distinct features of these waters, aiding in understanding source authenticity and quality. Furthermore, our findings will provide a foundation for future research into the environmental impact of water production and transportation methods, aiding informed decisions regarding bottled water consumption.

## 2 Materials and methods

Several bottled water brands were obtained from a local supermarket at King Abdullah University of Science and Technology (KAUST) in October of 2020 (Supplementary Table 1). Additional samples were collected and examined between October of 2020 and April of 2022. Although flavored water and vitamin-enriched water have recently grown in popularity (Rani et al., 2012), they were not analyzed for the purpose of this study due to the nature of additives. The samples were compared through physical, chemical, and microbiological analyses (Table 1). A minimum of 2 replicates for each bottled water were analyzed, but more bottles were examined if needed. Additionally, tap water from six households in KAUST, where water is provided from a local reverse osmosis (RO) desalination plant with a daily output of 40,000 m^3^ (Belila et al., 2016), were also collected and examined for chemical and microbiological composition in comparison to bottled water samples. Tap water samples were collected in sterile 50 mL tubes after flushing the taps for 5 min. Samples were maintained at approximately 20°C during transportation (less than 10 min) and immediately stored at 5°C upon arrival at the laboratory. Analysis preparations commenced within 1 h of sampling.

**TABLE 1:** Summary of experiments conducted with the associated bottled water sample selections.

Bottled water features explored	Method	Brands investigated
Chemical composition	Ion chromatography, inductively coupled plasma-optical emission spectroscopy	All brands
Microbiological content	Flow cytometry	All brands
Bottle material	Flow cytometry	Prominent brand with plastic and glass versions
Microbial growth potential (MGP)	Flow cytometry	Select brands of contrasting types of water (mineral vs. purified vs. sparkling)
Microbial community composition	16S rRNA gene sequencing	Prominent mineral water brands

### 2.1 Physical and chemical analysis

The composition label components of all bottled water brands were noted in Supplementary Table 2. The samples were analyzed soon after purchasing and were stored at 21–23°C in their original containers. A portable pH meter (WTW™ ProfiLine™ pH 3310) was used to measure pH for each sample. For sparkling water samples, the first stable value was promptly recorded. This allowed for capturing the most accurate pH value before CO_2_ escaping after opening the bottle and potentially increasing the pH.

The undiluted drinking water samples were filtered through 0.22 μm syringe filters to measure ionic compositions. The ions fluoride (F^–^), chloride (Cl^–^), bromide (Br^–^), nitrate (NO_3_^–^), nitrite (NO_2_^–^), and sulfate (SO_4_^2–^) were measured using an ion chromatograph [Dionex SP ICS-1600 (IC)] equipped with the separation column Dionex IonPac AS15 RFIC (2 × 250 mm) at a set temperature of 30°C and flow rate of 1 mL/min. The system was calibrated using Dionex Seven Anion Standard II (Thermo Scientific). The elements sodium (Na), calcium (Ca), potassium (K), magnesium (Mg), iron (Fe), lead (Pb), nickel (Ni), zinc (Zn), aluminum (Al), and copper (Cu) were measured by inductively coupled plasma optical emission spectroscopy (5110 ICP-OES, Agilent Technologies). Quality control was performed between every ten samples using an ICP multi-element standard (CPAchem) with concentrations of one and ten parts per million (ppm). To visualize chemical composition differences among bottled waters, a principal component analysis (PCA) was processed using RStudio with the “FactoMineR” package.

### 2.2 Microbiological analysis

The bacterial cell contents in bottled water (at least 2 replicates) were measured on the day of collection by flow cytometry [BD Accuri C6 Flow Cytometer (FCM)]. To prepare for FCM, 693 μL of each sample was stained with 7 μL of SYBR Green I (100× concentration) to enumerate the total cells, while another 690 μL of each sample was stained with 7 μL of SYBR Green I (100× concentration) and 3 μL of propidium iodide (PI), to enumerate the intact cells, hereafter referred to as live cells. Samples were incubated for 10 min at 35°C, and then 200 μL of each was transferred into a 96 well plate. The data was analyzed using the BD Accuri C6 software, with the system parameters and electronic gating applied to select for each stain and separate positive signals from noise and sample background (Hammes et al., 2008; Prest et al., 2013). A sample of the gating applied is shown in the Supplementary Figure 7. MilliQ water (sterile, 0.22 μm filtered) was used as a blank between samples, and unstained samples were used as controls to rule out autofluorescence from the samples. For sparkling water samples, a small volume was dispensed into a separate sterile tube and shaken to hasten the bubbles release and prevent bubbles during analysis. The estimated time for the fastest release of bubbles from sparkling waters at 20°C is in the order of 10 min (Liger-Belair et al., 2015). To ensure consistent staining time and conditions for all samples, one sample (Evian) was analyzed at both the start and end of the 96-well plate used for FCM analysis. No significant difference was observed in the calculated cell concentrations between these two measurements, Samples’ results were compared using a Wilcoxon Rank-Sum Test when the data did not follow a normal distribution; otherwise, a *t*-test was used. Data were analyzed for normality using a Shapiro-Wilk test.

### 2.3 Microbial growth potential assessment

The bottled water samples subjected to microbial growth assessments were chosen based on their diversity and prevalence in the market (Supplementary Table 3). Special attention was given to the investigation of purified waters and mineral waters, specifically chosen for their contrasting cell concentrations. To this end, the sampling tube was inserted into the newly opened bottle which was sealed and punctured with a small hole (to avoid vacuum during sampling). After each sample’s first measurement at room temperature, the bottles were incubated in a water bath at 30°C and cell concentration was measured over time and recorded using an automated online FCM system (BD Accuri C6 Flow Cytometer & onCyt OC-300) (Supplementary Figure 1). The temperature was selected based on optimal mesophilic growth (Sato et al., 2020). The measuring time between each sample was 20 min. In the case of sparkling water samples, the sampling tube was inserted well below the water surface where bubbles rise, and no bubbles were expected at 30°C or after 20 min (Spagnolie et al., 2024). All samples were treated in the same manner. The minimum incubation period was one week, and the maximum allowable period was two weeks due to the limited volumes. A SYBR Green I staining solution (10,000× concentration) was used to measure the total cells over time. The protocol followed and the electronic gating for BD Accuri C6 software were according to Hammes and Besmer (2018). The software used for data acquisition included the OnCyt software, which directs instructions to the OnCyt system to function, and the BD Accuri C6 software, which receives the data through remote interfacing and measures the total cell numbers. These experiments were parallel to the previous microbiological analysis and included new bottled water samples. The initial cell values of the bottles used in the online growth potential tests were comparable to other bottles used in offline analysis.

In order to identify microbiological changes over time and between varying types of water, phenotypic fingerprints collected from the FCM were used to analyze Beta-diversity through performing a principal coordinate analysis (PCoA) using the Bray-Curtis dissimilarity (Hasanin et al., 2023; Props et al., 2018).

Before starting incubation experiments, approximately 30 mL from each bottled water sample was collected to measure the waters’ total organic carbon (TOC) using a TOC analyzer (Shimadzu TOC-V CPH Total Organic Carbon Analyzer). The extracted volumes were filtered using a pre-cleaned 0.22 μm syringe filter to remove microbial content and prevent it from altering the TOC results; thus, it can be referred to as dissolved organic carbon (DOC) or TOC that passed through a 0.22 μm filter. The calibration was set from 1 to 10 ppm. Quality control of 1 ppm TOC was placed after groups of 5 samples and at the end. The concentrations of TOC were also used to calculate and predict the extent of microbial growth that may occur when TOC is bioavailable (Lesaulnier et al., 2017); Typically, 1 μg of carbon may yield between 4 × 10^6^ and 20 × 10^6^ cells/mL (Prest et al., 2016b). The apparent maximum growth rate was calculated from the slope of the exponential growth phase (Hagen, 2010).

### 2.4 Microbial community analysis

Microbial community analysis was performed on prominent mineral water brands previously assessed for microbial growth potential (Supplementary Table 4). The samples (2.6 to 4 L), which were unopened separate bottles from previous analyses, were filtered through a Millipore™ 0.22 μm mixed cellulose esters membrane filter using a Millipore™ filtration system at 720 mbar vacuum. The membranes were stored at −80°C until DNA extraction was performed.

Each sample was filtered in two separate instances; once before incubation to detect the inherent microbial communities present, and again after incubating the same volume of bottles at 30°C. Samples were incubated for the same time length it took to observe microbial growth during previous incubation experiments. For instance, if maximum bacterial growth was achieved on day 7 for Sample X, then Sample X will be incubated for 7 days before DNA extraction. Samples were filtered when the microbial growth was expected to peak, to collect the microbial communities that were active throughout incubation, and before the stationary phase (Supplementary Table 4).

The DNA extraction was performed using the MP Bio FastDNA™ Spin Kit for Soil following the manufacturer’s protocol with a modified bead beating time of 40 s repeated twice to improve cell lysis. Samples were placed on ice for two minutes after each cycle (Gonzalez-Gil and Holliger, 2011). The DNA was extracted from the membrane filters, and the concentrations of DNA obtained were quantified using Qubit 3.0 Fluorometer Qubit assays with high sensitivity calibrations of 0.2–100 ng. Before sequencing, samples below 2 ng of DNA were concentrated using SpeedVac. The DNA concentrates were kept at −80°C until ready for microbial community analysis.

For microbial community analysis, the samples were analyzed using 16S rRNA gene amplicon sequencing targeting the bacterial variable regions V3-4, using the forward [341F] CCTACGGGNGGCWGCAG and reverse [805R] GACTACHVGGGTATCTAATCC primers (Patel, 2001). Following amplification, the sequencing libraries were prepared following the Illumina protocol (Illumina, 2015). The purified sequencing libraries were pooled in equimolar concentrations and diluted to 2 nM. Then, the amplicons were sequenced with a MiSeq (Illumina, USA) instrument and using a MiSeq Reagent kit v3 (Illumina, USA). Supplementary material contains a detailed description of the protocol. For the sequence analyses, the resulting forward and reverse reads were trimmed for quality using Trimmomatic v. 0.39 (Illumina, 2015), and then the forward and reverse reads were merged. All the quality-filtered sequences were then collapsed into a set of unique reads (i.e., de-replicated) which were then clustered into operational taxonomic units (OTUs). Taxonomy was assigned using the RDP classifier (Bolger et al., 2014) and the SILVA database (Wang et al., 2007). The results provided were analyzed using RStudio with the ampvis package (Quast et al., 2012). For further details, see Supplementary material. Sequencing reads were deposited in the NCBI Short-Read Archive under the BioProject number PRJNA1116707.

## 3 Results and discussion

### 3.1 Brands and varieties of bottled drinking waters available in the market

In total, 19 distinct brands of bottled drinking water were identified and categorized into five types of bottled water based on their labels (Supplementary Table 1): purified drinking water, natural mineral water, spring water, artesian water, and sparkling water. For this study, spring water is classified as mineral water since several brands labeled as natural mineral waters were sourced from springs (e.g., Evian and Volvic). Some brands offered water in either plastic or glass containers, or both, and in some cases, the same brand had different water sources or bottling locations (e.g., Nestle Pure Life). In one case, two separate brands were bottled at the same location (i.e., Tamimi Markets and Hana). Some bottled waters, such as Voss and Berain, had still water as well as carbonated still water (i.e., sparkling water). Henceforth, samples from brands with more than one bottling location or bottle material shall be identified in this study as: Brand name- location abbreviated or brand name- bottle material as P or G (P for plastic and G for glass).

Purified bottled waters, which contain water that undergoes a form of treatment and disinfection (Ferrier, 2001; Tarver, 2008; Hunter, 1993), were primarily sourced locally in Saudi Arabia from desalinated water, well water, or groundwater. Nestle and Aquafina bottled waters, two of the largest drinking water producers worldwide, were produced through RO, a treatment process commonly used in seawater desalination to provide potable water in Saudi Arabia (Shomar and Hawari, 2017). In contrast, mineral, artesian, and sparkling bottled waters were mostly imported from Europe, with over 60% of the brands being non-local. The environmental impact of transporting bottled water, particularly in terms of CO_2_ emissions, may be significant, as imported natural waters contribute more due to long-distance transportation compared to locally produced purified waters (Gleick and Cooley, 2009). Although purification methods like RO can also contribute to CO_2_ emissions for locally produced purified bottled water, the energy costs of long distance transportation may still be greater (Gleick and Cooley, 2009). Nonetheless, evaluating the degree of environmental impact related to transportation, treatment, and production methods of different bottled waters based on the receiving location (i.e., Saudi Arabia) must be studied.

Another distinction among bottles was their production and expiration dates (Supplementary Table 1). Purified waters were valid for one year, sparkling waters for two years, and mineral waters for one to two years. Although water itself can have an infinite shelf life, its quality and taste can degrade over time due to chemicals leaching from containers or other processes that can occur during prolonged storage (Posnick and Kim, 2002). This suggests that expiration dates pertain to the packaging, potentially to protect consumers from any adverse effects from long-term packaging (Posnick and Kim, 2002; Raj, 2005).

### 3.2 Chemical composition of bottled waters

The average composition of components indicated on the bottled water labels were categorized based on water type (Table 2). Components commonly listed for all samples included bicarbonate, sulfate, chloride, fluoride, nitrate, calcium, magnesium, sodium, and potassium. Additionally, some components were unique to certain water types, such as silica (all types excluding purified), bromate (purified only), nitrite (mineral only), carbonate (purified and sparkling), and iron (all types excluding artesian). The full list of values listed per sample is noted in Supplementary Table 2. Comparing still waters, purified waters displayed higher levels of chloride (avg. 27 mg/L) and sulfate (avg. 32 mg/L) over mineral waters. This is potentially attributed to the chemical pretreatment processes of purified water (Ahmad and Azam, 2019). Most bottled mineral waters exhibited elevated mineral concentrations; mineral water is characterized by total dissolved solid content above 250 ppm (Dijkstra and de Roda Husman, 2023). However, it was observed that sparkling water samples possessed the highest overall mineral content, likely added to enhance or modify their taste (WHO, 2022). In brands offering both still and sparkling water, notably Berain and Voss, distinct mineral compositions were observed. Sparkling Berain had slightly higher nitrate (0.3 mg/L) and chloride (37 mg/L) levels compared to its still counterpart (0.1 and 35–36 mg/L), along with some variations in calcium, magnesium, and sodium, resulting in unique overall chemical profiles. Similarly, sparkling Voss had significantly higher sodium levels (122 mg/L) than still Voss (3.8 mg/L). These differences in mineral content can justify categorizing sparkling water separately from other types.

**TABLE 2:** Range of mineral composition values for all components as stated on the bottled water composition labels for purified, mineral, artesian, and sparkling water.

Parameter (mg/L)	Purified	Mineral	Artesian	Sparkling	Guideline limits	
	min–max	min–max	min–max	min–max	WHO	SASO
Carbonate	< 1	n/a	0*	< 4*	n/a	n/a
Bicarbonate	1.3–50	106–360	152*	60–1,250	n/a	n/a
Sulfate	9–85	4–< 28	1–2.1	2.1–401	250^†^	150
Chloride	< 1–50	2.6–15	5.5–9	5.5–49.6	250^†^	150
Fluoride	0.8–1	< 0.1	0–0.13	< 0.2–1.2	1.5	0.8–1.5
Nitrate	< 1–3	0.5–7.3	1*	0.3–7.3	50	50
Nitrite	n/a	0.01*	n/a	n/a	3	n/a
Calcium	< 1–27	12–80	3.7–18	3.7–166	n/a	200
Magnesium	2.3–21.1	8–26	0.9–15	0.9–80	n/a	150
Sodium	< 5–17	2–< 15	3.8–18	9.6–180	200^†^	100
Potassium	< 1–8	0.7–6	5*	0.9–2.1	n/a	n/a
Iron	< 0.1	< 0.1*	n/a	0.01*	0.3^†^	0.3
Bromate	< 0.01	n/a	n/a	< 0.005*	0.01	0.01
Silica	n/a	6.9–32	93*	27*	n/a	n/a
TDS	105–155	141–345	36–222	310–1,100	600^†^	500
Total hardness	41–< 90	130–170	106*	39–60	n/a	n/a
pH	6.5–8.5	7–8	6.6–7.7	5–6	6.5–8.5^†^	6.5–8.5

*Value was only noted for one sample.

^†^Value is suggested based on acceptability aspects of drinking water (i.e., taste, odor, and appearance), although no limit is strictly defined. n/a: parameters were not listed for type of bottled water or health guidelines were not specified. The following international and local upper limit values and pH ranges were listed in comparison: World Health Organization (WHO, 2022; WHO, 2021) and Saudi Arabian Standards Organization (SASO) (Ghrefat, 2013).

Most bottled water samples follow standards set by the WHO and the local Saudi Arabian Standards Organization (SASO). As could be expected, the two distinct brands (i.e., Tamimi Markets and Hana) that were bottled at the same location contained the same chemical compositions, and the brand with bottles from two separate bottling locations (i.e., Tamimi Markets) contained differing chemical compositions.

The range of pH values for each type of water was: 6.5–8.0 for purified water, 6.6–7.7 for artesian water, 7–8 for mineral water, and 5–6 for sparkling water. The experimental pH values for all bottled water samples were within ± 10% of the label. Sparkling waters had the lowest pH values due to carbonation. All samples adhere to the WHO recommended pH range of 6.5–8.5, which the WHO considers an aesthetic quality rather than a health-based guideline for drinking (WHO, 2022). Due to differences in pH and chemical composition, sparkling water samples were grouped separately from still water types. Notable variations were also observed within brands offering both sparkling and still water (Supplementary Table 2). Furthermore, lower pH can significantly influence microbial community compositions in various environments (Santini et al., 2022; Rousk et al., 2010; Fierer and Jackson, 2006).

The ion concentrations of most bottled waters from different sources followed the guideline limits set by the WHO, however, few exceeded the limit (Supplementary Table 5). For instance, sparkling water sample S.Pellegrino had ∼60% greater SO_4_^2–^ concentration than the limit set by WHO and SASO. However, this limit is mainly set due to potential taste impairments (WHO, 2022). The measured concentrations of Cl^–^, F^–^, and NO_3_^–^ were within guideline limits for all samples. The concentrations measured in tap water samples were very close in value to each other, which was expected since it is distributed through a small distribution network within a small community. Deviations between label and experimental values ranged from 0% to over 200% with most differences being minor; label values primarily serve as average compositions (Supplementary Table 5). The frequency and extent of testing conducted by manufacturers on bottled water batches are unclear, potentially resulting in increased variability among individual bottles and discrepancies between label and experimental values.

As with ion concentrations, there were variations in most cases between labels and experimental element values (Supplementary Table 6). Samples generally comply with regulations set by the WHO and SASO, and harmful trace elements were undetected or measured at levels far below the guideline limits. Sparkling water samples were found to have the highest overall concentrations of analytes. Some elements present in water constitute essential minerals required for daily diets, such as Fe, Na, Ca, K, and Mg, though the amounts needed vary. There are no guidelines or health concerns by the WHO, but higher concentrations of these elements may affect the taste or acceptability of water (WHO, 2022). High Na concentrations were found among sparkling and tap water samples, which can provide a distinct flavor or unacceptable taste (Doria, 2006; Shomar and Hawari, 2017). Hence, the higher concentrations of some elements may contribute to the different flavors of water which may be preferable, or unappealing, to individuals. Regarding healthiness, higher mineral concentrations in drinking water do not supplement dietary needs enough to be of significance [ National Research Council (US) Safe Drinking Water Committee, 1980; Jern, 2022; WHO, 2022].

**FIGURE 1:**
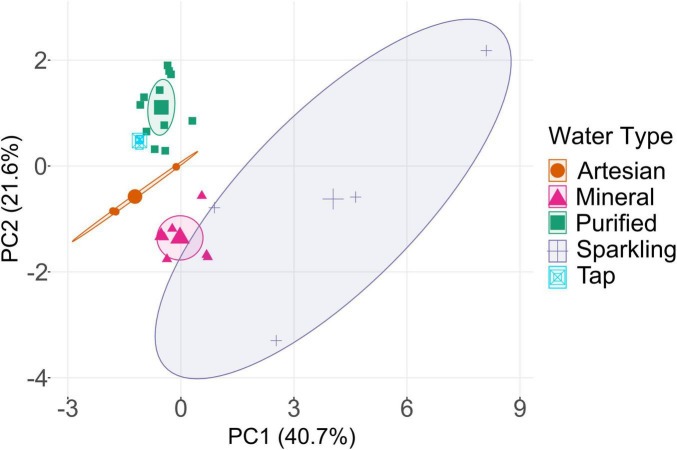
Principal component analysis (PCA) based on major ions in bottled waters (Supplementary Tables 5, 6). The distinct concentrations for each water type are reflected in their grouping. Tap water is clustered closest to purified bottled water, indicating similar chemical constituents. The central symbols represent the centroids of the respective ellipses, helping to visualize the central location of each group relative to the others.

The distribution of bottled waters based on chemical composition (Ca, Na, Mg, K, Cl^–^, F^–^, SO_4_^2–^, NO_3_^–^, and NO_2_^–^) is represented in a PCA plot (Figure 1). The PCA demonstrates that there is a clear distribution among samples based on the type of drinking water. Among the four water types, purified and mineral waters exhibited close clustering within their respective groups, yet they remained distinctly separate from each other. Sparkling and artesian bottled waters also had some similarities, but they were more widely distributed in the PCA plot across PC1, showing greater variations within sparkling and artesian water types. There were also fewer samples of sparkling and artesian waters than other types, and a greater variety in the mineral content of each sample. Although some sparkling water brands are simply carbonated versions of still water, they were distinguished from their still counterparts by higher levels of sodium (Voss), nitrite (Voss and Berain), and nitrate (Berain), leading to distinct groupings in the PCA analysis despite sharing the same source. Based on the PCA, tap water, which is treated via RO, displayed clustering alongside purified bottled water samples, indicating a close similarity in their chemical compositions and treatment processes; thus, highlighting the unique chemical signature imparted by RO treatment. Given the undisclosed treatment methods for several brands, it is reasonable to infer in this case, purified samples are synonymous with RO treatment. In regions like Saudi Arabia, where tap water is often treated by RO, purified bottled water and tap water may undergo comparable treatment processes, thereby blurring the distinction between them. Indeed, purified water can originate from the same source of municipal water (Studlick and Bain, 1980; Gleick and Cooley, 2009; Al-Zahrani et al., 2017). This multivariate analysis may prove valuable in identifying unknown drinking water samples by associating them with established categories, or potentially replicating unique chemical features characteristic of specific water types.

### 3.3 Difference in number of microbial cells between naturally sourced and purified waters

**FIGURE 2:**
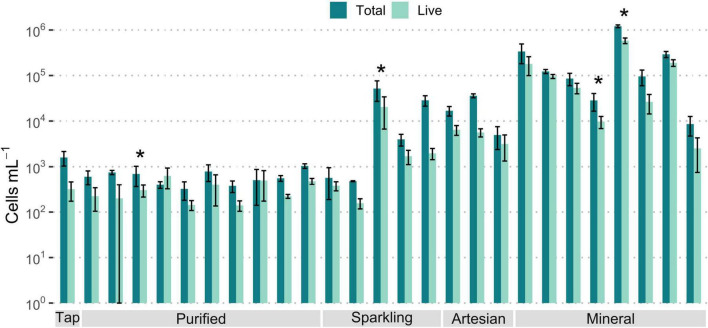
Average concentrations of microbial cells, in logarithmic scale, measured through flow cytometry for all bottled water samples, categorized by type. A large percentage of bacterial cells are live for most samples. Error bars indicate standard error. 10 ≥ *n* ≥ 3. Asterisks (*) denote *n* = 2.

FCM analysis results show great variation for microbial cell counts among the different bottled waters (Figure 2). The total microbial cells ranged from less than 10^3^ cells/mL in purified waters to 10^6^ cells/mL in mineral waters. Most natural mineral waters had the highest cell numbers among samples, while purified waters had the lowest numbers. Artesian waters had around 10^3^- 10^4^ cells/mL, while sparkling waters contained up to 10^5^ cells/mL. The number of live microbial cells were less than the total cells at varying degrees for different samples, but a large percentage of total cells for most samples were intact.

Most purified bottled water samples contained total cell counts below 10^3^ cells/mL, and live cell counts below 500 cells/mL (Supplementary Figure 2). Tap water samples had comparably low cell numbers, which approached the detection limit of the instrument. The detection limit of the FCM varies and ranges from as low as 32 to 200 cells/mL (Gillespie et al., 2014; Hammes et al., 2008); Thus, the purified bottled water values of about 200 cells/mL were near the detection limits as reported for drinking water. Purified drinking waters, as well as tap water, go through steps of purification and disinfection, and these processes are effective enough to remove or destroy most microbial cells in water (Bitton, 2014; Rosenberg, 2003), resulting in the low microbial values observed. Membrane filtration methods, such as RO, can lower microbial cell concentrations by over 99.5% of cells and reduce assimilable organic carbon (Prest et al., 2016b; Sousi et al., 2020). Based on chemical and microbiological analysis, it is reasonable to infer that most purified bottled water samples have undergone RO treatment.

Unlike purified bottled water, mineral water samples exhibited total cell counts up to 10^6^ cells/mL, and most samples consisted of high intact cell counts above 10^4^ cells/mL (Supplementary Figure 3). Greater microbiological cell numbers were expected with mineral waters due to their lack of extensive treatment to maintain their natural state, thus preserving their inherent microbial flora (Bitton, 2014). The natural occurrence of microbes in bottled waters, which is a flagship characteristic of mineral water, is not of health concern as they are inspected for known pathogens and fecal coliforms to ensure safety for consumers (Fewtrell et al., 1997; Leclerc and Moreau, 2002). Most mineral waters with higher cell contents originated from France. However, Tannourine, originating from Lebanon, was the mineral water with the highest cell counts among all waters investigated. This may suggest that its water sources may harbor a richer microbiological environment than other locations. The microbial cell concentrations in drinking water typically range from 10^3^ to 10^6^ cells/mL (Prest et al., 2016b), which is comparable to the range of cells found in the tested bottled waters. As with the chemical composition PCA (Figure 1), unknown bottled waters may be identifiable through their microbial composition, as larger cell numbers can be attributed to natural waters contrary to treated purified waters.

Artesian samples contained total cells ranging from 490 cells/mL to 4.25 × 10^4^ cells/mL, resembling purified waters on the lower end and mineral waters with low cell numbers on the higher end (Supplementary Figure 4). Artesian water is groundwater under pressure that flows naturally from a confined aquifer to the surface, while mineral water is not pressurized underground and is characterized by richness in minerals (Bullers, 2002; Tarver, 2008). Artesian water, comparable to mineral water in its natural preservation, may be exhibiting lower cell numbers than mineral water due to its inherent natural filtration process, as the confined high pressure within the aquifer filters the water through porous rock and sand, effectively serving as a natural water filtration system capable of reducing microbe presence (Dijkstra and de Roda Husman, 2023; Jain et al., 2019).

For sparkling water samples, the majority had lower total microbial cell concentrations between 10^3^ and 10^4^ cells/mL (Supplementary Figure 5). Out of the five samples, two are naturally carbonated, and the rest were artificially carbonated. The naturally carbonated samples had an average of 4 × 10^3^ and 5.1 × 10^4^ total cells/mL. The artificially carbonated samples had average total cell counts ranging from < 10^3^ to 2.8 × 10^4^ cells/mL. The carbonated purified water Berain and the carbonated artesian water Voss contained cell numbers in the range of their still water counterparts. The number of cells present in sparkling natural waters may be less than still mineral water samples due to the low pH and carbonation limiting microbial content (Bitton, 2014; Rosenberg, 2003).

**FIGURE 3:**
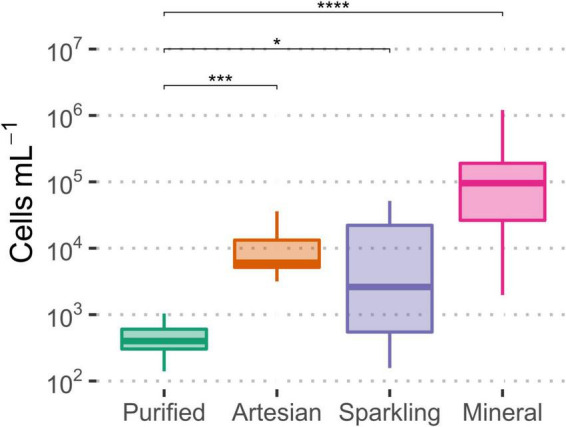
Significant differences observed in microbial cell concentrations among the four types of bottled water, relative to purified water, as measured by flow cytometry. Significance levels were determined via Wilcoxon test (*p ≤ 0.05; ***p ≤ 0.001; ****p ≤ 0.0001).

Among the types of bottled waters, there were significant differences between bottled purified water cell concentrations and other types of water (Figure 3). The largest significant difference was observed between mineral and purified waters. Typically, purified water would contain less microbial cells due to undergoing further treatment, such as membrane filtration or disinfection, whereas natural water, untouched by such processes, would likely contain higher cell numbers (Bitton, 2014); natural filtration does not reduce the number of microbial contents as effectively as membrane filtration. Ultimately, the microbiological content for all bottled waters was within the expected range for drinking water.

### 3.4 Microbial growth responses during incubation for different types of water

**FIGURE 4:**
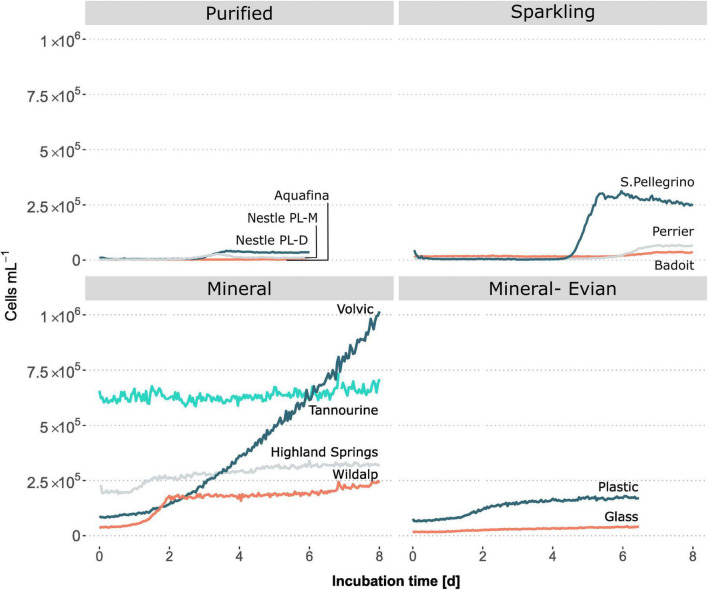
Microbial growth as a function of incubation time at 30°C among different types of bottled water and bottle materials, as measured by online flow cytometry. The growth patterns vary among different types of bottled water and between mineral water bottled in plastic versus glass.

The following analysis sought to investigate the potential and extent for mesophilic growth at optimal temperature (30°C) in different types of bottled water. Findings revealed that the extent and rate of microbial growth varied among the different bottled water samples, with most showing some degree of growth during incubation at 30°C (Figure 4 and Table 3). On the other hand, when stored at 5°C, plastic-bottled mineral water Evian maintained a consistent cell count for 18 days, ranging from 8.0 × 10^4^ to 8.9 × 10^4^ cells/mL. This highlights temperature’s impact on microbial growth potential.

**TABLE 3:** Total organic carbon measurements, cell growth observed and maximum growth rates during incubation of bottled drinking water.

	Sample	TOC pre-incubation (mg/L)	Initial cell numbers at *T* = 0 (cells/mL)	Max growth achieved (cells/mL)	Max readily bioavailable carbon utilized (mg/L)	Apparent max growth rate (h^–1^)	Doubling time (h)
Purified	Aquafina	0.08	2.522 × 10^3^	9.657 × 10^3^	0.002	0.101	6.9
	Nestle PL Dammam	0.07	2.388 × 10^3^	3.545 × 10^4^	0.01	0.075	9.2
	Nestle PL Madinah	0.08	2.418 × 10^3^	2.786 × 10^4^	0.01	0.074	9.4
Mineral	Evian in glass	1.15	1.675 × 10^4^	4.087 × 10^4^	0.01	0.010	69.3
	Evian in plastic	0.52	6.573 × 10^4^	1.686 × 10^5^	0.03	0.021	33.0
	Highland Springs	0.30	1.928 × 10^5^	3.543 × 10^5^	0.04	0.014	49.5
	Tannourine	1.05	6.564 × 10^5^	7.731 × 10^5^	0.03	na	na
	Volvic	0.30	8.403 × 10^4^	2.176 × 10^6^	0.52	0.009	77.0
	Wildalp	0.67	3.859 × 10^4^	4.064 × 10^5^	0.09	0.058	11.9

na indicates no apparent growth observed. The potential maximum concentration of readily bioavailable carbon utilized was estimated based on the TOC pre-incubation, cell growth achieved, and the yield of 4 × 10^6^–20 × 10^6^ cells per 1 μg of carbon (Prest et al., 2016b).

After incubation, the sample with the maximum cell content was Volvic, a natural mineral water, while the sample with the least maximum growth was Aquafina, an RO purified water sample (Figure 4). Relative to their initial cell counts, overall microbial growth in purified bottled waters was much less than mineral waters. Studies have shown that some mesophilic microbial cells can grow and even remain viable for over 11 months in natural mineral bottled waters (Gonzalez et al., 1987). Indigenous microbial populations found in natural mineral waters have been documented, through HPC, to undergo rapid growth and reach concentrations of 10^4^–10^7^ cells/mL within a few days after bottling, even when stored at ambient temperatures (Daood, 2008). However, it’s important to note that the HPC method can primarily detect the growth of bacteria capable of utilizing the carbon present in the culture media, typically of an easily biodegradable nature, rather than the more representative intrinsic carbon content present in the water, as demonstrated in this study.

During the incubation of bottled mineral waters, all samples had diverse microbial growth responses (Figure 4). One sample, Highland Springs, experienced growth initially but stabilized afterward. Alternatively, Wildalp experienced a diauxic growth with a second growth phase occurring after the first week of incubation; Extension of Wildalp analysis duration was possible, as it was continued for a second week where the diauxic growth phase was noted along with changes in microbial compositions, after the first stationary phase, per FCM plots (Supplementary Figure 6). The microbial cells present during the second growth phase may have either belonged to a different community capable of utilizing a new carbon source or adapted by modifying their metabolic processes to utilize an alternative carbon source (Chu and Barnes, 2016).

For sparkling water samples, the sample (Badoit; pH = 6) with the highest initial microbial cell count had the lowest growth rate and lowest maximum growth when compared with the other samples (Figure 4 and Table 3). In contrast, the sample (S.Pellegrino; pH = 5.2) with the lowest initial cell counts among sparkling water samples had the highest growth rate and maximum growth. The low pH of sparkling waters and the presence of carbon dioxide could have restricted microbial growth initially (Daood, 2008; Bitton, 2014). Over time, the slow release of carbon dioxide and the reduction of pH acidity may have allowed for microbial growth to occur. Due to the FCM setup for continuous measuring, the effect of pH is unknown. However, one might consider for future studies using a pH sensor interfaced with a computer for online measurements; this would make tracking periodic changes in pH possible.

Purified bottled water samples had the lowest cell concentrations and microbial growth overall (Figure 4). While samples started with similar cell numbers, the two Nestle Pure Life samples underwent growth about two days later, while Aquafina remained stable until a minor growth occurred five days later. Despite water treatment processes ensuring purification and absence of microbes, it is common for microbial regrowth to occur after distribution reaching concentrations up to 10^4^–10^5^ cells/mL (Gatza et al., 2013). Aquafina was processed through RO, while Nestle Pure Life samples were sourced from desalinated water, which also undergoes RO. Drinking waters purified through RO exhibited limited amount of growth due to having the least number of microorganisms and the least amount of DOC, as shown in the following section. Municipal water supplies processed via RO and extensive treatments could be investigated for microbial growth and stability to compare its quality to that of purified bottled waters. Purified bottled waters had the shortest shelf life of one year, however, based on the inherent concentration of microbial cells and microbial growth potential in comparison to other types of waters, it may be able to sustain stability for a longer period. This suggests that the bottle validity periods may not be considering microbial quality stability or may be arbitrary altogether.

### 3.5 Effect of total organic carbon concentrations available on the extent of microbial growth potential

The presence of bioavailable TOC can impact the potential for microbial growth observed in bottled water samples previously (Figure 4). Therefore, TOC was measured pre-incubation for each sample (Table 3). The TOC ranged from 0.07 to 0.08 mg/L for purified waters, and from 0.30 to 1.15 mg/L for natural mineral waters. On average, purified waters contained > 80% less TOC than mineral waters. The minimal microbial growth observed in purified water correlates with its lower TOC content compared to mineral water samples. Additionally, natural mineral waters exhibited the highest cell concentrations, while treated purified waters displayed the lowest, reflecting the differences in organic carbon content. The TOC values for sparkling water were not measured due to high concentrations of CO_2_. It would be necessary to remove all CO_2_ to obtain proper TOC measurements.

Based on TOC available pre-incubation, some samples did not reach the expected maximum growth potential (Table 3). Despite the recorded TOC concentrations, the biodegradability and type of carbon source in each sample is unknown as it is a complex mixture (Lam et al., 2007). Although Tannourine had a high amount of TOC, as well as the most microbial cells overall, there was no growth observed. It is possible that the organic carbon present may not be readily biodegradable (Frimmel, 1998; Sulzberger and Durisch-Kaiser, 2009), as indicated by the estimated readily bioavailable organic carbon based on the extent of microbial growth achieved, which shows that only a small fraction of TOC may have been biodegradable in most samples. One mineral water sample, Volvic, may have been able to utilize all the TOC available. Examining the relationship between TOC and maximum cell growth reveals a general correlation for most samples, although two samples did not fit into this trend (Supplementary Figure 8). Volvic exhibited low TOC but high microbial growth, whereas Evian in glass had high TOC but low growth. These anomalies suggest that factors besides TOC concentrations, such as the nature of the organic carbon or other environmental conditions, may influence microbial growth potential.

Furthermore, several samples reached a stationary growth phase with relatively stable microbial cell numbers over time (Figure 4), suggesting that excess organic carbon was either no longer bioavailable or became inaccessible. Further growth may have also been prohibited, or limited, by the lack of inorganic nutrients (e.g., nitrogen, phosphorous) or trace elements (e.g., iron) (Prest et al., 2016b). In any case, the higher microbial growth observed in mineral over purified waters corresponds to its difference in TOC, although its biodegradability is unknown. Classifying the biodegradable carbon available in bottled water may aid in further identification of its microbiological characteristics (Lesaulnier et al., 2017).

### 3.6 Comparison of microbial growth potential between plastic and glass bottles containing mineral water from the same source

An additional incubation experiment was performed to compare the effects of plastic and glass bottles containing the same sample on microbial concentrations and growth. There was a clear growth contrast between the same mineral water (i.e., Evian) bottled in glass and plastic (Figure 4). While Evian in glass reached a maximum growth of 4.087 × 10^4^ cells/mL, Evian in plastic reached 1.686 × 10^5^ cells/mL at double the growth rate (Table 3).

**FIGURE 5:**
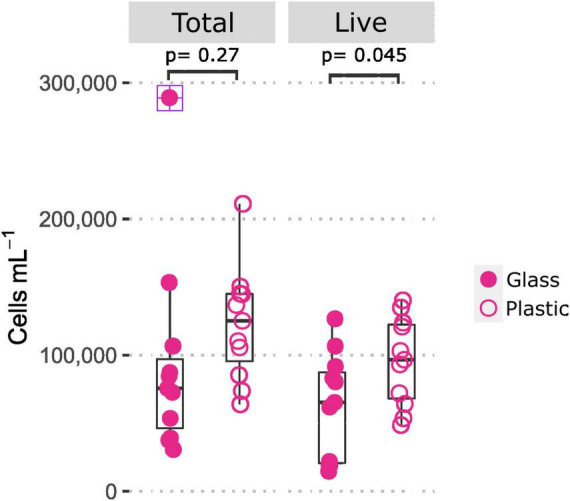
Comparison of total and live microbial cell concentrations in mineral water Evian bottled in glass and plastic, indicating a difference in live cell counts (*t*-test, *p* < 0.05). Each point denotes one replicate (*n* = 11). The plus symbol indicates an outlier value in the total cells for glass.

To further investigate the apparent differences in cell numbers between plastic and glass bottled Evian, offline FCM measurements were assessed with separate bottles in a parallel analysis and combined with previous microbial cell count results (Figure 5). The results suggest that there was a difference in the amount of live cells/mL between glass and plastic bottles (*t*-test, *p* = 0.045) (Benjamin and Berger, 2019). The amount of total and live cells for plastic bottle samples ranged from 0.639 to 2.11 × 10^5^ cells/mL and 0.487–1.402 × 10^5^ cells/mL, respectively. Whereas the total and live cells for glass bottles ranged from 0.307 to 2.891 × 10^5^ cells/mL and 0.148–1.267 × 10^5^ cells/mL, respectively. The upper range value for total cells in glass is over 1.5 times higher than the interquartile range (Figure 5).

While sourced from the same origin, samples in glass bottles had lower initial cell concentrations than in plastic. Although the glass and plastic bottled samples were purchased on the day of experimentation, the bottling date differed between samples. Due to the nature of collecting water from a natural source, microbial concentrations may vary over time. Plastic bottles have been shown to promote greater bacterial growth than glass bottles (Bischofberger et al., 1990; Bitton, 2014). Bacterial growth can likely occur from additives in plastics productions (Sheridan et al., 2022) and can even lead to biofilm formation on microplastics which can be detected in bottled waters (Maharjan, 2024). Also, plasticizers, which are chemicals added to promote plasticity that may leach into the water, may have some effect on microbial growth in plastic bottles. Some types of marine bacteria have been shown to degrade plasticizers (Wright et al., 2020), and one study found that organic carbon seeping from plastic influenced freshwater biofilm formation (Neu et al., 2018). In order to rule out the effect of different packaging on microbial content, an experiment can be designed to concurrently fill plastic and glass bottles from the same drinking water source to investigate how their microbiological qualities develop over incubation time (Nadreen, 2021). Nevertheless, more data is needed with a variety of bottled water types in glass and plastic to fully understand the impact of container material on microbial growth in bottled water. Future experiments should control for variables such as bottling date and source variation by concurrently filling plastic and glass bottles from the same water source.

### 3.7 Change in microbial communities during the growth period as revealed by FCM fingerprinting

Through FCM, the spectrum of microbial communities can be identified through “fingerprinting.” The stained microbial cells gated within the dot plots depict unique fingerprints representing the distinct microbial populations in each sample (Gatza et al., 2013). It is also able to discern minute changes in the community structure through microbial cluster separations based on fluorescence intensity and variations of cells with low nucleic acid (LNA) and high nucleic acid (HNA) content (Hammes and Besmer, 2018; Prest et al., 2013).

**FIGURE 6:**
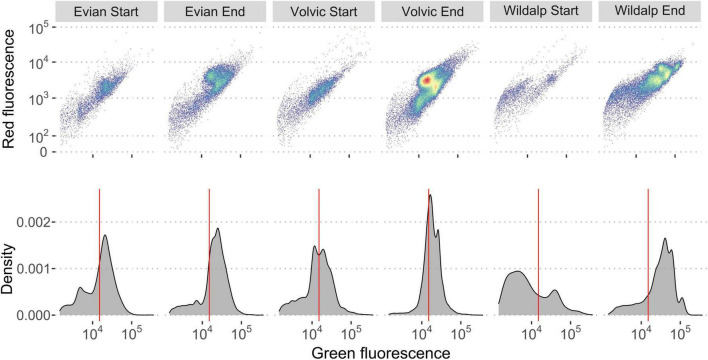
Flow cytometry histogram and dot plots demonstrate differences among three plastic-bottled mineral water samples at the start (day = 0) and end (day = 6) of an incubation period at 30°C through fingerprinting. The dot plots illustrate only the gated region. Electronic gating was applied based on previous study protocols (Hammes et al., 2008) and a sample of the gate is shown in the Supplementary Figure 7. The red line separates the high nucleic acid (HNA) and low nucleic acid (LNA) cells. Distinct changes are observed in cell clustering and the distribution of HNA and LNA cells between the start and end of incubation. Changes in cell numbers are detailed in Table 3.

Fingerprint variations during incubation can aid in recognizing shifts in microbial populations. Changes in “microbial fingerprints” were observed over time, coinciding with microbial growth during incubation (Figure 6). Each mineral bottled water sample exhibited changes in microbial content and unique fingerprints, as observed in the plots. Samples also had shifts in microbial cells distributions from LNA to HNA after incubation. Highland Springs’ microbial community shifted to a higher percentage of HNA cells after incubation. Wildalp had the greatest shift of HNA cells during incubation, increasing from 31.2 to 75.8% after incubation. Figure 6 illustrates how the microbial composition changes based on the flow cytometric signal. The scattering of microbial cells create fingerprints that are unique identifiers for different water samples (Gatza et al., 2013). Ultimately, incubating bottled water samples at 30°C may advance microbial growth as well as alter the initial microbial community composition, which can be observed through FCM data.

**FIGURE 7:**
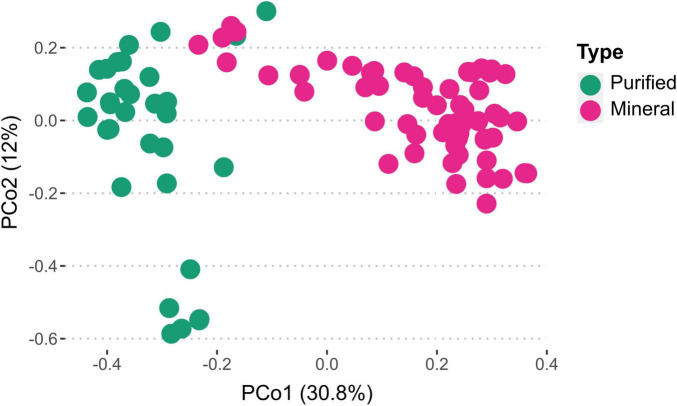
Ordination of phenotypic fingerprints measured by flow cytometry in purified and mineral bottled waters, illustrating the distinct separation of bacterial communities between the two water types. Cytometric fingerprint data points were collected every 24 h for three purified and five mineral water samples during the weeklong incubation period at 30°C as part of the online microbial growth potential tests.

Analyzing microbial communities via fingerprints obtained from FCM data can aid in contrasting purified water against other samples. Cytometric fingerprints obtained from online FCM analysis are distinct for purified and mineral bottled waters (Figure 7). Through phenotypic fingerprints, one can analyze clustering of microbial communities and compare any shifts between samples (Hasanin et al., 2023), such as the changes that occurred after incubation of bottled waters (Supplementary Figure 9). For a better resolution, microbial community analysis can be accomplished through DNA extractions and gene sequencing.

### 3.8 Microbial community analysis and most abundant genera

To investigate the bacterial community composition and the extent of community changes in bottled waters, 16S rRNA genes were sequenced from samples before and after incubating water bottles at 30°C. The most prevalent mineral bottled waters in the market were chosen in plastic containers to explore their microbial communities. As the purified water sample Nestle Pure Life is an RO purified drinking water, the DNA extracted was below the detection limit, and thus, was not analyzed. Although downstream processing of samples yielding DNA contents below the limit of detection is possible, higher PCR bias can occur when the initial DNA template concentration is below detection limits (Polz and Cavanaugh, 1998). Retrieving 5 ng of DNA from the purified water sample Nestle Pure Life would have required filtering over 200 L, considering the average total cell count and DNA concentration per cell (Button and Robertson, 2001). Achieving this would be quite challenging, especially given that extraction efficiency is seldom 100%.

**FIGURE 8:**
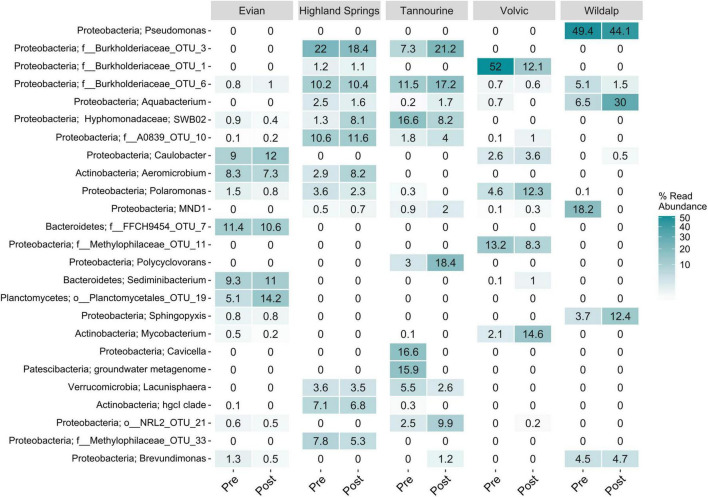
Microbial community analysis showing the 25 most abundant genera found in five mineral bottled water samples, showcasing shifts in microbial communities after a 6–7-day period of incubation at 30°C. “Pre” denotes microbial communities present before incubation, while “Post” denotes those present after incubation.

For natural mineral waters, the 25 most abundant genera and their relative abundance among samples before or after incubation are identified in Figure 8. The identification of microbial cells for most was limited to genera, and few were limited to phyla. Wildalp had the lowest microbial diversity but contained some of the most abundant genera overall. Proteobacteria was the prominent phylum present in Wildalp and most other samples. Proteobacteria are a ubiquitous phylum of gram-negative bacteria frequently found in drinking water and in tap water after disinfection (Vaz-Moreira et al., 2017). However, the possible implications on human health is not well studied due to the phylogenetic diversity of the ubiquitous Proteobacteria (Vaz-Moreira et al., 2017). Of the Proteobacteria phylum, *Aquabacterium* was found in all samples, except Evian, and was the most abundant genus in Wildalp post incubation at 30% abundance. *Aquabacterium* is commonly found in natural mineral water after bottling and in freshwater biofilms; so, biofilm presence in bottling plants could be a potential origin (Loy et al., 2005). Another genus detected was *Pseudomonas*, which was only found in Wildalp and was the most abundant genus at 49.4% without incubation and 44.1% after incubation. *Pseudomonas* is an abundant genus of Proteobacteria found in drinking water, and some species may pose health risks to humans (Vaz-Moreira et al., 2017; Mena and Gerba, 2009). Nonetheless, the *Pseudomonas* involved is unknown, and there exist many nonpathogenic species (Stolp and Gadkari, 1981; Divya et al., 2018). Assuming adherence to strict quality control and guidelines, it is unlikely that any of the samples contain a bacterium that may pose a health hazard.

Of the other phyla discovered: Actinobacteria, found in Evian, Highland Springs, and Volvic; Bacteroidetes, present in Evian and Volvic; and Planctomycetes, detected exclusively in Evian, are ubiquitous in aquatic environments and often possess important biogeochemical roles (Wiegand et al., 2018; Freitas et al., 2012). Bacteroidetes, as with Proteobacteria, are among the most abundant types of bacteria found in drinking water (Vaz-Moreira et al., 2017). Additionally, Patescibacteria were identified only in Tannourine, while Verrucomicrobia were found in both Highland Springs and Tannourine. Planctomycetes, which nearly tripled in abundance in Evian to 14.2% after incubation, can be opportunistic pathogens and affect human hosts (Wiegand et al., 2018), and *Mycobacterium*, detected in Volvic and increasing to 14.6% abundance after incubation, includes species that are pathogenic and can be found in potable water (Niva et al., 2006; Pfaller et al., 2022). Verrucomicrobia is detected in most aquatic environments and categorized as the sixth most abundant bacterial phylum in the ocean (Freitas et al., 2012).

Shifts in microbial community abundances were observed after incubating bottled water samples at 30°C. Although the microbiological composition was affected during incubation, the degree of microbial diversity in examined drinking water samples was not greatly altered. While some changes in abundances were minor, other concentrations shifted considerably. For Evian, *Limnobacter* and *Chitinophagaceae* decreased in abundance from 20 to 4.1% and from 16.5 to 0.2%, respectively. *Limnobacter* has been found in various environments including seawater and volcanic deposits, and only a few species have been characterized (Chen et al., 2016). For Tannourine, Proteobacteria *Cavicella* and a Patescibacteria found in groundwater in a metagenome study were no longer detected after incubation. Patescibacteria are prevalent in groundwater, sediments, and other water environments (Tian et al., 2020), whereas *Cavicella* is a novel genus with species found in mineral water aquifers (França et al., 2015). Little is known about their functions or potential pathogenicity. In Wildalp, *Sphingopyxis* increased in abundance to 12.4% after incubation. *Sphingopyxis* strains, found in various environments, have potential for bioremediation and biodegradation of harmful environmental contaminants (Sharma et al., 2021). Proteobacteria SWB02, part of the oligotrophic-adapted Hyphomonadaceae family, was detected in Evian, Highland Springs, and Tannourine at varying concentrations before and after incubation. This genus has also been found in slow sand filters used for drinking water production (Abraham and Rohde, 2014; Bai et al., 2023). Another notable taxon found exclusively in Tannourine was *Polycyclovorans*, which increased from 3 to 18.4% abundance after incubation. This marine genus includes species with the potential to degrade aromatic hydrocarbon (Gutierrez et al., 2013).

In the end, the most abundant bacterial taxa in the investigated bottled water samples correspond to bacteria commonly found in natural aquatic ecosystems, such as springs, aquifers, and volcanic deposits. Nonetheless, further investigation into the types of species present is necessary. Implementing new methods to achieve long read sequencing would enable the identification of organisms at the species level, thereby facilitating the detection of organisms of concern (Curry et al., 2022; Tedersoo et al., 2021). Additionally, it would enhance the exploration of species attributes and provide insights into the compositions and origins of bottled drinking water.

**FIGURE 9:**
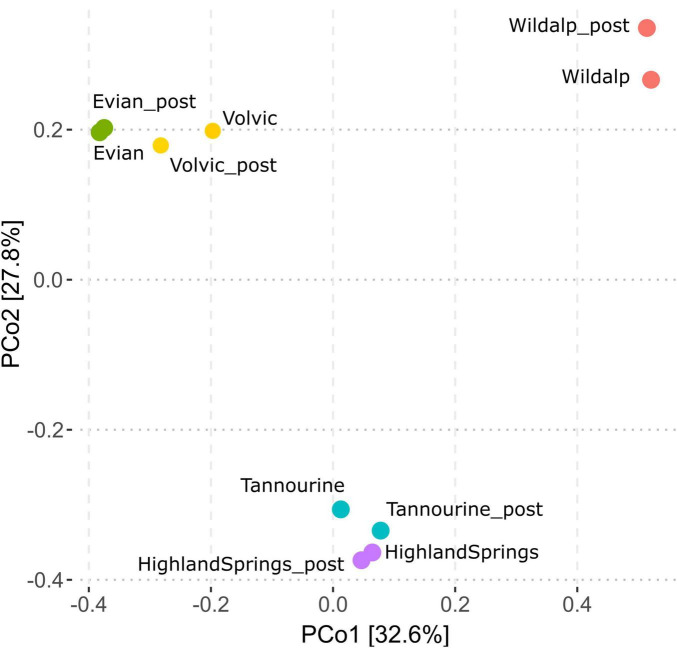
Principal coordinates analysis (PCoA) plot based on operational taxonomic units (OTUs) from microbial community analysis, comparing samples before and after incubation and showing the distribution of bottled waters relative to their country of origin. Sample names suffixed with “_post” indicate the community distribution post-incubation. Post-incubation samples exhibit similar distribution patterns to their non-incubated counterparts.

The microbial communities present in bottled waters have led to a distinct characteristic distribution based on incubation status and bottled water brand (Figure 9). Samples with greater shifts in microbial community post-incubation were distributed slightly displaced from their non-incubated counterparts (i.e., Volvic, Tannourine, and Wildalp); however, in general, post incubation samples remained adjacent to those without incubation. This suggests that the microbial composition is unique to the bottled water source and not considerably influenced by incubation; thus, the inoculum source is an important factor in shaping the community.

Ultimately, incubation did not significantly influence the microbial community structure for bottled natural mineral water samples. Furthermore, no two bottled water brands possessed the same microbial structure. Previous studies using PCR along with denaturing gradient gel electrophoresis also showed that distinct bottled mineral waters contain its own bacterial community, creating a unique molecular fingerprint (Bitton, 2014).

## 4 Conclusions

In this study, we performed comprehensive chemical and microbiological analyses to compare purified, mineral, artesian, and sparkling bottled drinking waters from 19 brands. Each water type exhibited distinct chemical compositions, with significant differences that highlight the potential for categorization based on these variations. Microbiological characteristics also varied, with purified waters showing the lowest microbial cell concentrations and most mineral waters showing the highest, indicating the efficacy of purification treatments and the natural preservation of mineral waters. Additionally, results convey a difference in intact microbial cell content between mineral waters in plastic and glass bottles, implying an influence of bottle material, though more research is needed to confirm this as a general trend.

Incubation experiments at temperatures optimal for mesophilic growth revealed varying degrees of microbial growth responses among different types of bottled waters, influenced by microbial cell concentrations and dissolved organic carbon levels. Mineral water bottled in plastic appeared to have higher microbial growth than that in glass, suggesting potential differences that may warrant further examination.

DNA sequencing revealed the most abundant genera in five mineral bottled waters, indicating innocuous taxa commonly found in natural aquatic environments and that are potentially source specific. Furthermore, shifts in microbial community distributions were observed following incubation. Flow cytometric fingerprinting emerged as a valuable tool for microbial evaluations when DNA sequencing was not feasible.

Ultimately, the combination of the chemical and microbial signatures found in bottled waters exhibit a unique profile, which can hold significances for the verification of source and quality, thus attributing their authenticity. Future studies should explore quantifying the energy costs associated with the transportation and production of local purified and imported bottled waters to identify options with minimal environmental impact, given that all options already adhere to drinking water health guidelines.

## Data Availability

The datasets presented in this study can be found in online repositories. The names of the repository/repositories and accession number(s) can be found in this article/Supplementary material.
