# Supplementary material for: The unique chemical and microbiological signatures of an array of bottled drinking water
Source: Front Microbiol. 2024 Sep 16;15:1441142. doi: 10.3389/fmicb.2024.1441142 (PMC11439718; doi:10.3389/fmicb.2024.1441142)
Supplement: Supplementary file 1 [file Data_Sheet_1.PDF]

Supplementary Material

The unique chemical and microbiological signatures of an array of bottled drinking water

Yasmeen M. Nadreen*, Johannes S. Vrouwenvelder, Pascal E. Saikaly, Graciela Gonzalez-Gil

*** Correspondence:** Yasmeen M. Nadreen: yasmeen.nadreen@kaust.edu.sa

1 Supplementary Figures and Tables

1.1 Supplementary Figures

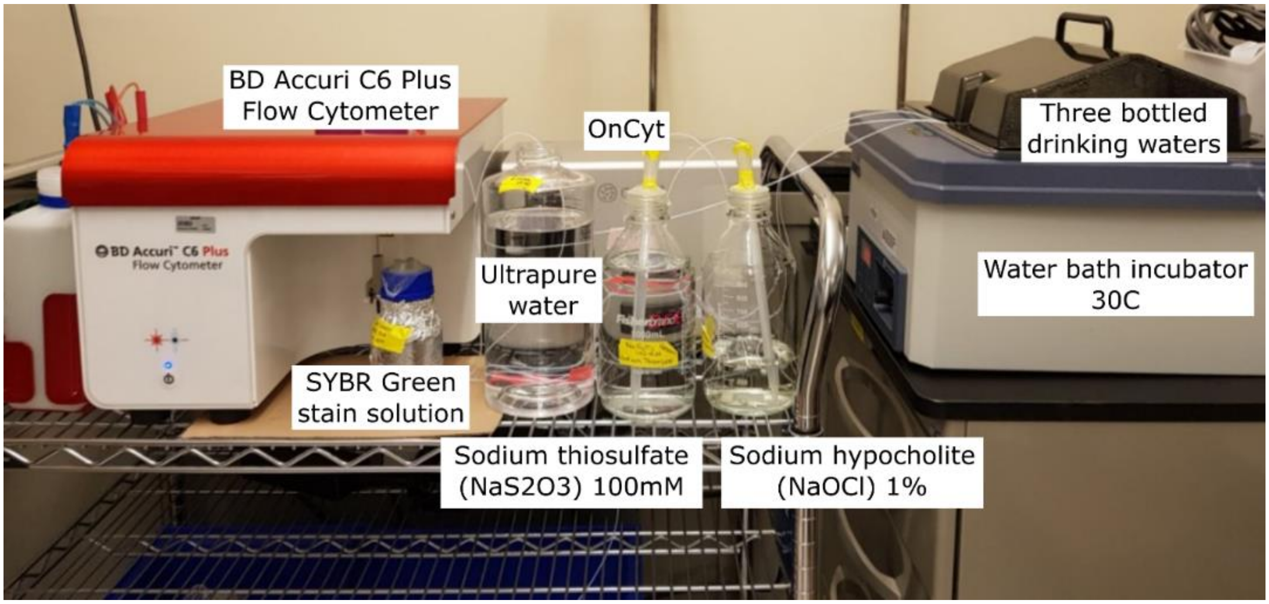

Figure S1: Online flow cytometry system consisting of OnCyt, BD Accuri C6 Plus, and water bath incubator for assessing the microbial growth of bottled drinking waters. Microbial cells in samples are stained with SYBR Green. NaS2O3, NaOCl, and ultrapure water were, sequentially, used to clean the system in between samples.

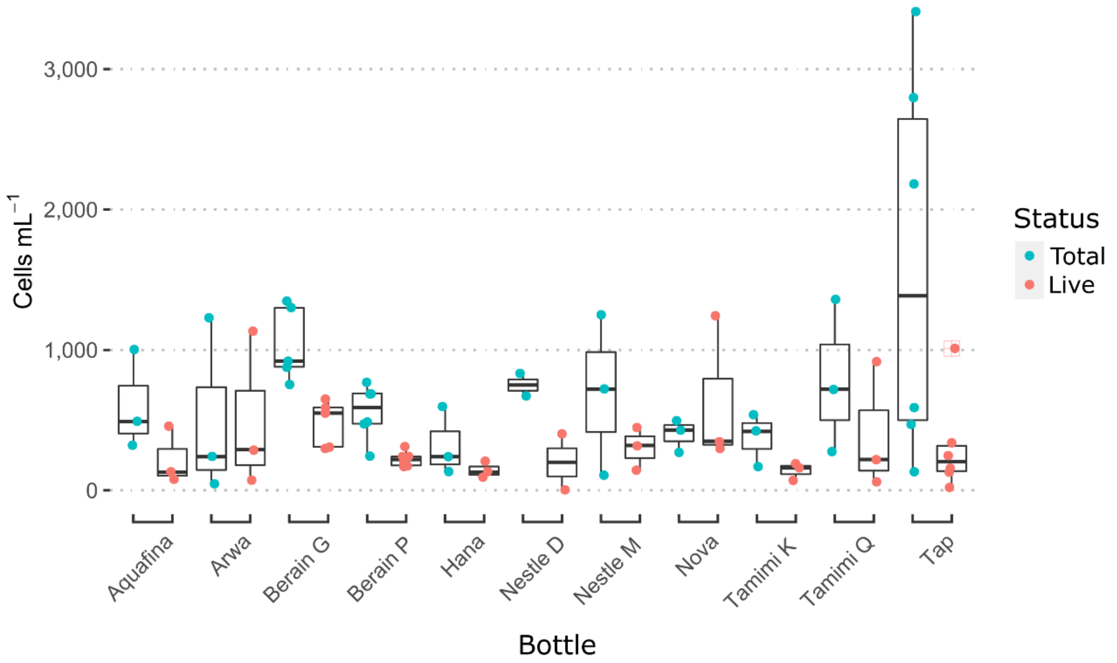

Figure S2: Purified water and tap microbial cell concentrations as measured through flow cytometry with at least three replicates per sample. Each point denotes one replicate.

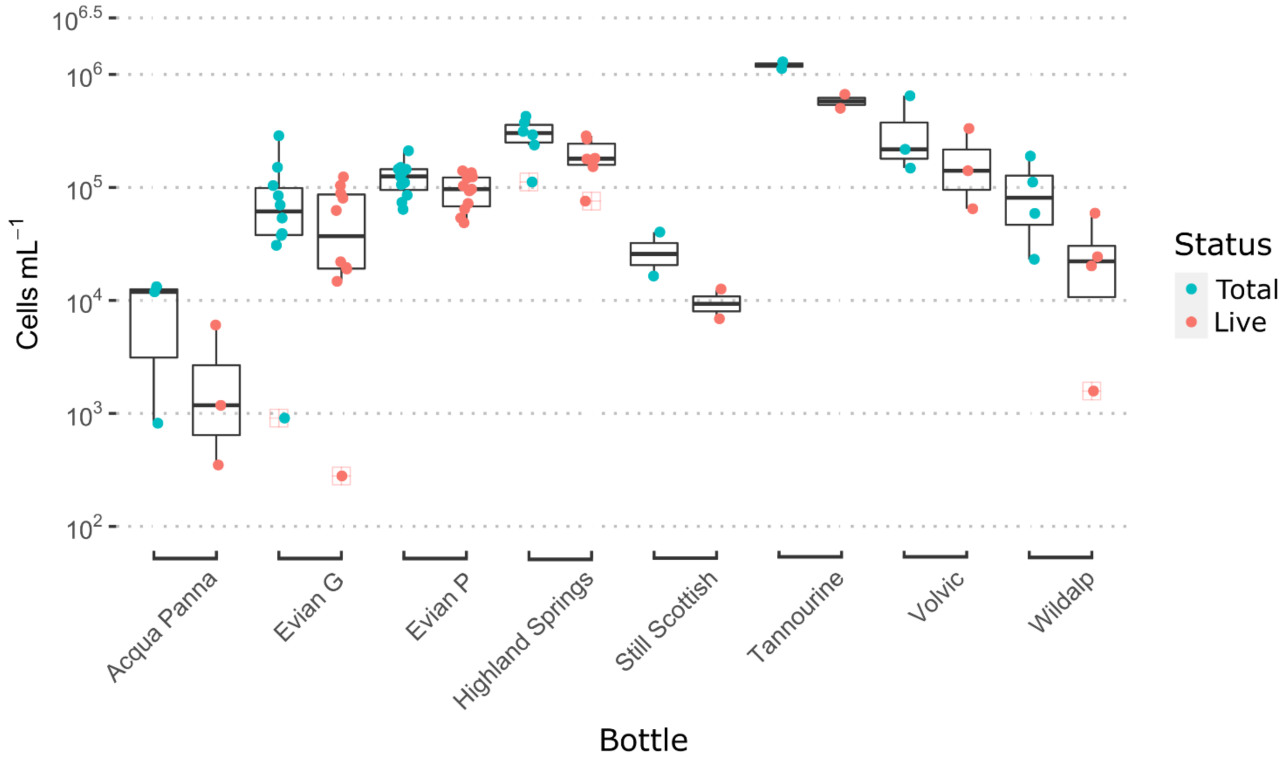

Figure S3: Mineral water microbial cell concentrations, in logarithmic scale, as measured through flow cytometry with at least two replicates per sample. Each point denotes one replicate. Cell values are noted for samples in the low range box plots.

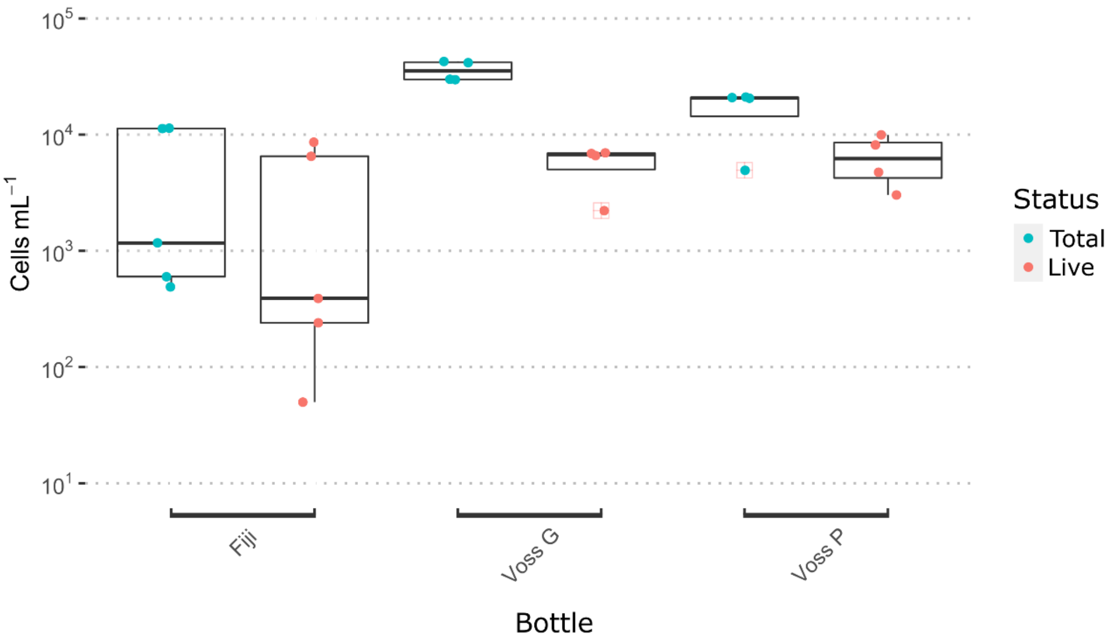

Figure S4: Artesian water microbial cell concentrations, in logarithmic scale, as measured through flow cytometry with at least three replicates per sample. Each point denotes one replicate.

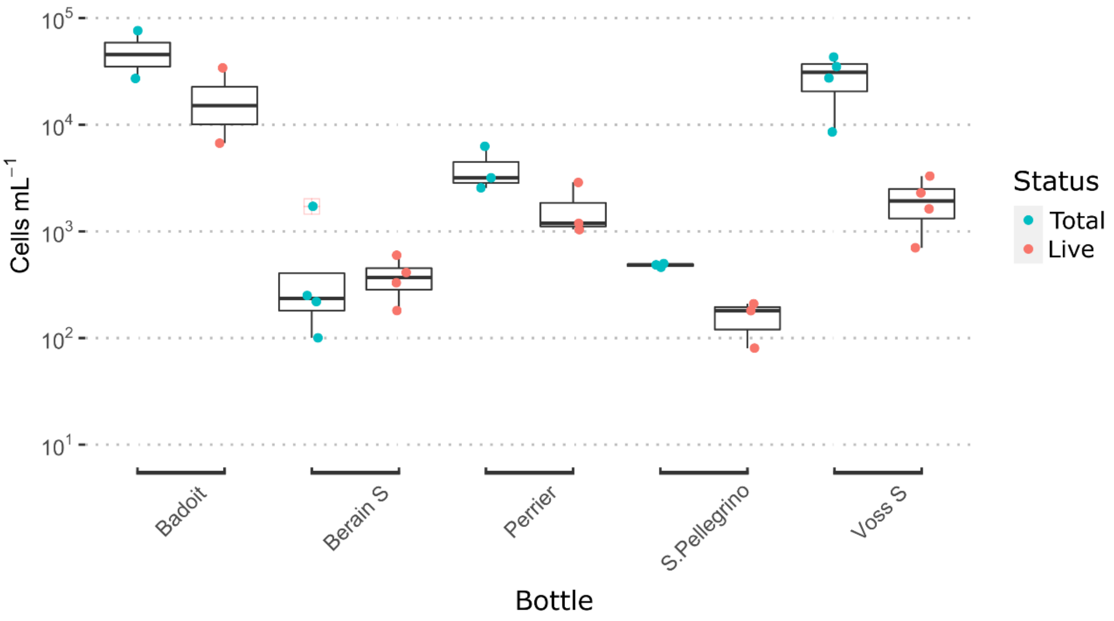

Figure S5: Sparkling water microbial cell concentrations, in logarithmic scale, as measured through flow cytometry with at least two replicates per sample. Each point denotes one replicate.

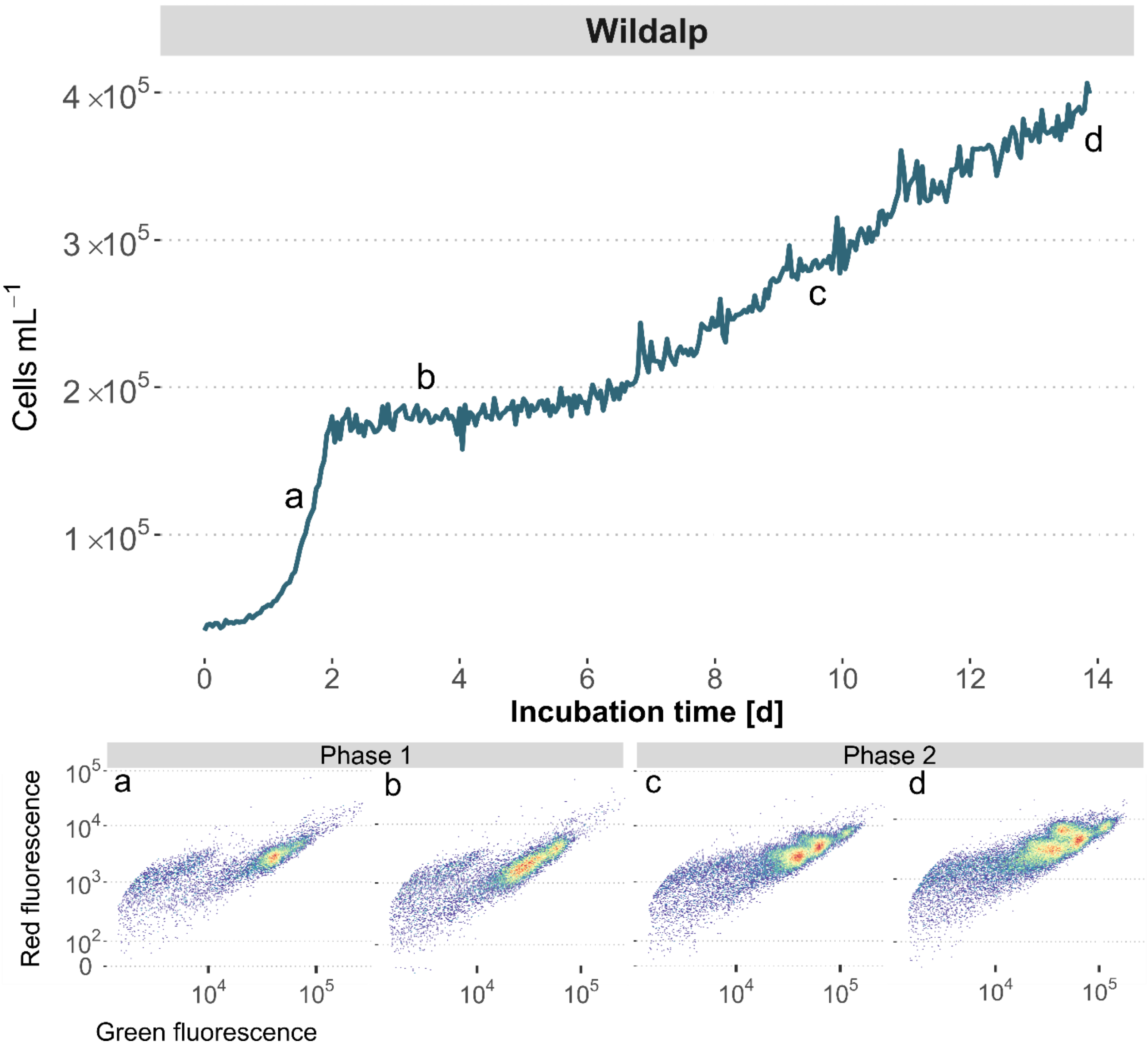

Figure S6: Growth potential test for mineral bottled water sample [Wildalp], with the entire incubation run in two weeks at 30°C, illustrating a diauxic microbial growth phase occurring during the second week. Online flow cytometry plots, or fingerprints, showcase the changing microbial community distribution after a second growth phase. The sample's volume limited the incubation period. The dot plots illustrate only the gated region.

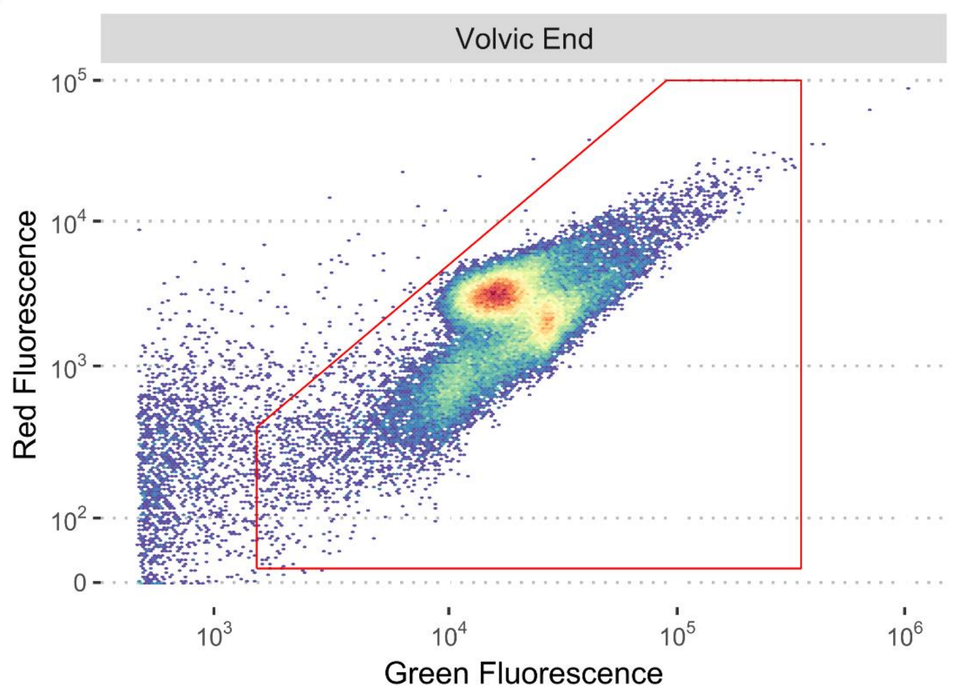

Figure S7: A flow cytometry dot plot indicating the electronic gating used to analyze the data (Hammes et al., 2008). This dot plot presents the mineral water sample Volvic at the end of incubation during the microbial growth experiments, also seen in Figure 6.

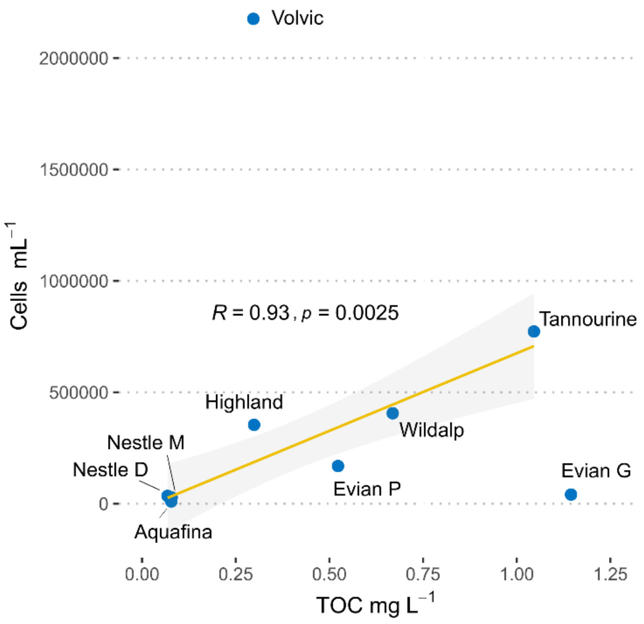

Figure S8: Linear regression showing the correlation between total organic carbon (TOC) and the maximum cell growth in samples analyzed during microbial growth potential tests (Chapters 3.4 – 3.5). Most samples fit the correlation (R^2= 0.93), indicating a relationship between TOC and microbial growth. However, Volvic, with low TOC but high growth, and Evian, with high TOC but low growth, did not conform to the linear trend.

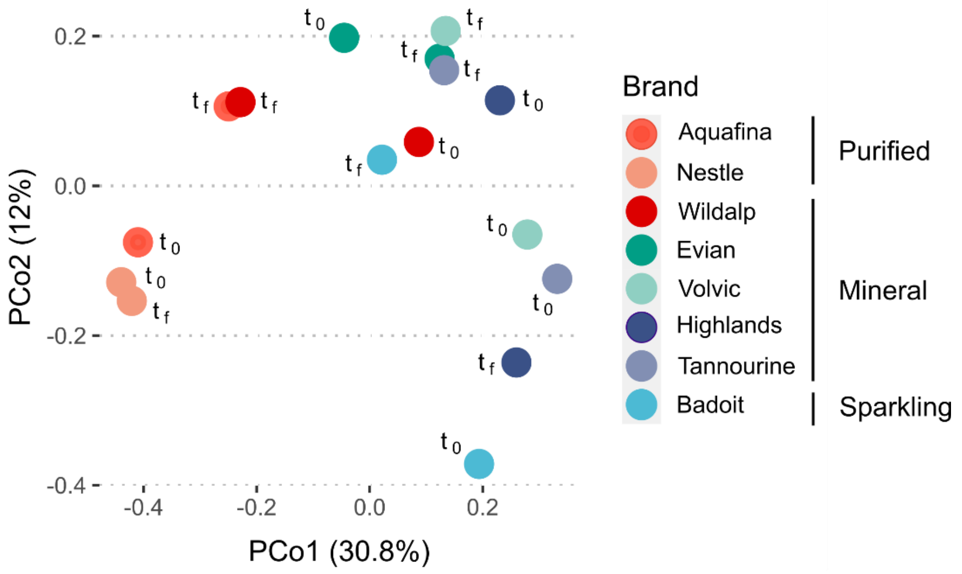

Figure S9: Ordination of phenotypic fingerprints measured by flow cytometry in bottled waters before incubation (t_0) and after incubation for several days when an apparent maximum growth was reached (t_f). Variations are noted between distribution of purified bottled water samples clustering on one side, and mineral bottled water samples on the opposing side.

1.2 Supplementary Tables

Table S1: General information on all bottled water samples collected for analyses including brand name, bottle material, origin location, volume, and production and expiration dates as labeled on the bottles.

<table>
<tr><th>No. Brand</th><th>No. Bottles Overall</th><th>Brand Name</th><th>Bottle Material</th><th>Water Type</th><th>Water Source</th><th>Bottling Location, Country</th><th>Volume (mL)</th><th>Date: Production-Expiration</th></tr>
<tr><td>1</td><td>1</td><td>Acqua Panna</td><td>Glass</td><td>Natural mineral water</td><td>Toscana, Italy</td><td>Sanpellengrino S.p.A. Acqua Panna springs, Florence, Italy *</td><td>250</td><td>27/03/2019-01/03/2021</td></tr>
<tr><td>2</td><td>2</td><td>Aquafina</td><td>Plastic</td><td>Purified water</td><td>Groundwater</td><td>AlJomaih beverage bottling company.
Buraidah, KSA</td><td>330</td><td>28/07/2020-27/07/2021</td></tr>
<tr><td>3</td><td>3</td><td>Arwa</td><td>Plastic</td><td>Purified water</td><td>Well water</td><td>Saudi Coca cola beverage bottling company.
Sudair city, Riyadh, KSA</td><td>330</td><td>18/03/2020-18/03/2021</td></tr>
<tr><td>4</td><td>4</td><td>Badoit</td><td>Glass</td><td>Sparkling natural mineral water</td><td>Badoit source</td><td>SAEME. Saint Galmier, France</td><td>330</td><td>20/11/2018-19/11/2020</td></tr>
<tr><td rowspan="3">5</td><td>5</td><td rowspan="3">Berain</td><td>Plastic</td><td rowspan="2">Purified water</td><td>*</td><td>Berain company.
Jeddah, KSA</td><td>330</td><td>22/05/2020-22/05/2021</td></tr>
<tr><td>6</td><td>Glass</td><td rowspan="2">Well water</td><td rowspan="2">Berain company.
Riyadh, KSA</td><td>300</td><td>10/03/2020-10/03/2021</td></tr>
<tr><td>7</td><td>Glass</td><td>Sparkling water</td><td>750</td><td>23/12/2019-23/12/2020</td></tr>
<tr><td>6</td><td>8</td><td>Evian</td><td>Plastic</td><td>Natural mineral water</td><td>French Alps</td><td></td><td>330</td><td>01/10/2019-01/10/2021</td></tr>
</table>

No. Brand	No. Bottles Overall	Brand Name	Bottle Material	Water Type	Water Source	Bottling Location, Country	Volume (mL)	Date: Production-Expiration
	9		Glass			Cachat spring- S.A.E.M.E. Evian, France	330	17/07/2019-17/07/2021
**7**	10	Fiji	Plastic	Natural artesian water	Yaqara, Viti Levu, Fiji Islands	Natural waters of viti limited. Yaraqa, Fiji	500	10/12/2019-10/12/2020
**8**	11	Hana	Plastic	Purified water	Well water	Hana company for food industries. AlQassim– Buraidah, KSA	330	30/12/2019-30/12/2020
**9**	12	Highland Springs	Plastic	Natural mineral water	Drawn from organic land in Scotland	Highland spring Ltd. Perthshire, Scotland, UK *	500	06/02/2020-06/02/2021
**10**	13	Nestle Pure Life	Plastic	Purified water	Underground water or desalination water	Springs water factory co. Dammam, KSA	200	17/08/2020-16/08/2021
	14				Desalination water	Naqiah water factory co. Madinah, KSA	330	16/09/2020-15/09/2021
**11**	15	Nova	Plastic	Purified water	Underground well water– Saad City	Health water bottling company. Saad, KSA	200	unknown
	16				Well water– Nufoud Se'ed	HWB co. plant. Seed, KSA	330	22/03/2020-22/03/2021
**12**	17	Perrier	Glass	Natural mineral water & CO_2	Water captured at the Perrier source	N.W.S, SUD. Vergeze, France *	330	27/05/2020-01/05/2022
**13**	18	S.Pellegrino	Glass	Sparkling natural mineral water	San Pellegrino, Italy	Sanpellegrino S.p.A. San Pellegrino Terme, localita Ruspino, Italy *	250	09/07/2020-08/07/2022

No. Brand	No. Bottles Overall	Brand Name	Bottle Material	Water Type	Water Source	Bottling Location, Country	Volume (mL)	Date: Production-Expiration
**14**	19	Still Scottish Mountain Water by Sainsbury's	Plastic	Still mountain water, underground	Caledonian water-eastern edge of the Campsie Fells	Produced in the UK for Sainsbury's Supermarkets Ltd, London	500	n/a-07/01/2022
**15**	20	Tamimi Markets Bottled Drinking Water	Plastic	Purified water	Well water–Al Shadida Valley	Tania bottled drinking water factory. AlKharj, KSA	200	03/07/2019-03/07/2020
	21				Well water	Hana company for food industries. AlQassim– Buraidah, KSA	200	22/03/2020-22/03/2021
**16**	22	Tannourine	Plastic	Natural spring water	Daher & Sarkis Sources	Societe Libanaise des Souces des Eaux de Tannourine s.a.l Lebanon ⁎	330	09/03/2020-09/03/2021
**17**	23	Volvic	Plastic	Natural mineral water	French Volcanoes	Clairvic spring. Volvic, France ⁎	330	21/10/2019-21/10/2021
**18**	24	Voss	Plastic	Artesian natural mineral water	Artesian water	Voss production AS. Voss, Vantestrom, Norway ⁎	330	19/12/2019-19/12/2021
	25		Glass				375	11/7/2019-11/7/2021
	26		Glass	Sparkling water			375	28/11/2019-28/11/2021
**19**	27	Wildalp	Plastic	Natural spring water	Hochschwab Massif spring water-Styrian Alps/ Seisensteinquelle	Wildalpen Wasserverwertungs GmbH Sausenbach, Wildalpen, Austria	500	05/03/2019-05/03/2021

⁎ Denotes that water was bottled at the source, as stated on bottle label

Table S2: Average mineral composition for each bottled drinking water samples as stated on bottle labels.

		Average Composition (mg/L)															
	**Sample**	**CO^{2-}**	**HCO_3^-**	**SO_4^{2-}**	**Cl^-**	**F^-**	**NO_3^-**	**NO_2^-**	**Ca**	**Mg**	**Na**	**K**	**Fe**	**BrO_3^-**	**SiO_2**	**TDS**	**Total hard-ness**
Purified	Aquafina	<1.0	1.3	85	1.4	0.97	<0.50	*	<1.0	20	4	<1.0	0.02	*	*	105	83
	Arwa	<1	7.7	74.5	<1	0.8	<1	*	<1	21.1	3	<1	*	*	*	121	<90
	Berain glass	*	20	9	35	1	0.1	*	22	3	17	5	0.01	<0.005	*	155	65
	Berain plastic	*	50	9	35	1	0.1	*	22	3	17	5	0.01	<0.005	*	155	65
	Hana	0	18	28	32	1	0.2	*	21	9	<5	8	*	<0.005	*	120	84
	Nestle Pure Life Dammam	*	22	10	50	0.8	<1	*	27	2.3	9.5	0.2	<0.02	<0.010	*	120	76 mg/L as $CaCO_3$
	Nestle Pure Life Madinah	*	22	10	50	0.8	<1	*	27	2.3	9.5	0.2	<0.02	<0.010	*	120	78 mg/L as $CaCO_3$
	Nova	*	26	26	19	1	3	*	11	3.4	17	1.2	*	*	*	120	41
	Tamimi Markets AlKharj	<1	19	40	17	0.9	<1	*	10	10	10	<1	<0.1	<0.010	*	110	65
	Tamimi Markets AlQasim	0	18	28	32	1	0.2	*	21	9	<5	8	*	<0.005	*	120	84

Average Composition (mg/L)

	Sample	CO^{2-}	HCO_3^-	SO_4^{2-}	Cl^-	F^-	NO_3^-	NO_2^-	Ca	Mg	Na	K	Fe	BrO_3^-	SiO_2	TDS	Total hardness
Mineral	Acqua Panna	*	106	22	7.8	<0.1	2.9	*	32.2	6.6	6.5	0.8	*	*	6.9	141	*
	Evian glass	*	360	14	10	0.06	3.8	*	80	26	6.5	1	*	*	15	345	*
	Evian plastic	*	360	14	*	0.06	3.8	*	80	26	6.5	1	*	*	15	345	*
	Highland Springs	*	150	5.3	6.1	*	3.1	*	40.5	10.1	5.6	0.7	*	*	*	170	130
	Still Scottish	*	<240	<28	<11	*	<6	*	<55	<16	<15	<2	*	*	*	<255	*
	Tannourine	*	160	4	10	<0.1	0.5	*	50	13	4	1	<0.1	*	*	190	170
	Volvic	*	74	9	15	*	7.3	*	12	8	12	6	*	*	32	130	*
	Wildalp	*	170	*	2.6	*	*	0.01	45	13	2	1	*	*	*	*	*
Artesian	Fiji	0	152	1	9	0	1	*	18	15	18	5	*	*	93	222	106
	Voss glass	*	*	2.1	5.5	0.13	*	*	3.7	0.9	3.8	*	*	*	*	36	*
	Voss plastic	*	*	2.1	5.5	0.13	*	*	3.7	0.9	3.8	*	*	*	*	36	*

		Average Composition (mg/L)															
	**Sample**	**CO^{2-}**	**HCO_3^-**	**SO_4^{2-}**	**Cl^-**	**F^-**	**NO_3^-**	**NO_2^-**	**Ca**	**Mg**	**Na**	**K**	**Fe**	**BrO_3^-**	**SiO_2**	**TDS**	**Total hard-ness**
Sparkling	Badoit	*	1250	35	54	1.2	*	*	153	80	180	11	*	*	27	1100	*
	Berain sparkling	*	60	9	37	1.1	0.3	*	20	2.3	18	0.9	0.01	<0.005	*	170	60
	Perrier	<4	420	25.3	19.5	<0.2	7.3	*	150	3.9	9.6	<1	*	*	*	456	39
	S. Pellegrino	*	*	401	49.6	0.5	*	*	166	49.5	30	2.1	*	*	*	853	*
	Voss sparkling	*	*	2.1	5.5	0.13	*	*	3.7	0.9	122	*	*	*	*	310	*

TDS signifies total dissolved solids

* Denotes values that were not noted on labels

Table S3: Microbial growth potential experiments performed to analyze the effect of incubation at 30°C on the microbial content for different types of bottled water. Information of samples tested, including type of samples, volume of bottles, and production dates of bottles, is noted.

Incubation Experiment	Bottled Drinking Water Samples	Figure Labels	Volume (mL)	Dates on Bottles
**Mineral**	1. Highland Springs	-Highland Springs	500	12/04/20
	2. Tannourine	-Tannourine	330	09/03/20
	3. Volvic	-Volvic	330	28/08/19
	4. Wildalp	-Wildalp	500	05/03/19
**Sparkling**	1. Badoit	-Badoit	330	21/11/19
	2. Perrier	-Perrier	330	24/06/20
	3. S. Pellegrino	-S. Pellegrino	250	09/07/20
**Purified**	1. Aquafina	-Aquafina	330	26/09/20
	2. Nestle Pure Life-Dammam	-Nestle PL-D	330	10/12/20
	3. Nestle Pure Life-Madinah	-Nestle PL-M	330	22/06/20
**Mineral – Evian plastic vs. glass**	1. Evian in plastic	-Plastic	330	27/09/19
	2. Evian in glass	-Glass	330	30/08/19

Table S4: Details of bottled water samples filtered for DNA extractions. Microbial community analysis was performed on these select bottled drinking waters based on high cell concentrations measured through flow cytometry in prior experiments. The volumes of bottled waters filtered were determined based on the average DNA content of bacterial cells (Button et al., 2001). The approximate yield of DNA was calculated from the total number of cells determined through FCM. From that, an appropriate volume was estimated for extracting enough DNA (at least 5ng of DNA per mL), assuming 100% retention of cells in the filter used and 100% extraction efficiency.

**Sample**	**Total Volume Filtered (L)**	**Length of Incubation (days)**	**Dates Listed on Filtered Bottles**
**Evian**	3.3	0	27/09/2019
**Highland Springs**	3	0	12/4/2020
**Nestle PL (Dammam)**	7.6	0	10/11/2020, 01/11/2020
**Tannourine**	2.6	0	9/3/2020
**Volvic**	3.3	0	28/08/2019
**Wildalp**	4	0	05/03/2019, 27/03/2019
**Sample Post Incubation**	**Total Volume Filtered (L)**	**Length of Incubation (days)**	**Dates Listed on Filtered Bottles**
**Evian**	3.3	7	27/09/2019, 09/08/2019
**Highland Springs**	3	9	12/4/2020
**Tannourine**	2.6	9	10/09/2020
**Volvic**	3.3	15	28/08/2019
**Wildalp**	4	9	28/03/2019

Table S5: Concentrations of anions in bottled water samples measured through ion chromatography alongside pH measurements in comparison to the label values and to international and local standards: the World Health Organization (WHO, 2022) and Saudi Arabian Standards Organization (SASO) (Ghrefat, 2013). Percent deviation from label values is noted.

		**Concentrations in ppm**							
	**Brand name**	**SO_4^{2}**	**Cl^-**	**F^-**	**NO_3^-**	**NO_2^-**	**Br^-**	**BrO_3^-**	**pH**
**Guidelines**	**WHO limits**	**250**	**250**	**1.5**	**50**	**3**	*****	**0.01**	**6.5-8.5**
	**SASO limits**	**150**	**150**	**1.5**	**50**	*****	*****	**0.01**	**6.5-8.5**
**Purified**	Aquafina	85	1.4	0.97	<0.50	*	*	*	6.5
	Experimental value	79.9	0.3	1.3	0.0	0.3	0.008	ϕ	6
	% Deviation	-6%	-79%	35%	n/a	n/a	n/a	n/a	-8%
	Arwa	74.5	<1	0.8	<1	*	*	*	7
	Experimental value	68.9	0.4	1.3	0.0	0.3	0.0036	ϕ	7
	% Deviation	-8%	0%	65%	n/a	n/a	n/a	n/a	0%
	Berain glass	9	35	1	0.1	*	*	<0.005	8
	Experimental value	9.1	34.0	1.3	0.1	2.7	ϕ	ϕ	9.2
	% Deviation	2%	-3%	31%	0%	n/a	n/a	n/a	15%
	Berain plastic	9	35	1	0.1	*	*	<0.005	8
	Experimental value	10.8	39.6	1.4	0.0	2.3	ϕ	ϕ	7.9
	% Deviation	20%	13%	41%	n/a	n/a	n/a	n/a	-1%
	Hana water	28	32	1	0.2	*	*	<0.005	7.8
	Experimental value	25.8	32.9	1.3	0.0	1.0	0.016	ϕ	7.4
	% Deviation	-8%	3%	35%	n/a	n/a	n/a	0.0	-5%

		Concentrations in ppm							
	**Brand name**	**SO_4^{2}**	**Cl^-**	**F^-**	**NO_3^-**	**NO_2^-**	**Br^-**	**BrO_3^-**	**pH**
**Guidelines**	**WHO limits**	**250**	**250**	**1.5**	**50**	**3**	*****	**0.01**	**6.5-8.5**
	**SASO limits**	**150**	**150**	**1.5**	**50**	*****	*****	**0.01**	**6.5-8.5**
**Purified**	Nestle pure life-Dammam	10	50	0.8	<1	*	*	<0.010	7
	Experimental value	11.2	51.7	1.4	0.0	2.1	0.005	ϕ	7.2
	% Deviation	12%	3%	72%	n/a	n/a	n/a	n/a	3%
	Nestle pure life-Madinah	10	50	0.8	<1	*	*	<0.010	7
	Experimental value	6.2	51.9	1.2	0.0	1.9	ϕ	ϕ	7.2
	% Deviation	-38%	4%	52%	n/a	n/a	n/a	n/a	3%
	Nova	26	19	1	3	*	*	*	7.4
	Experimental value	36.8	18.8	1.1	2.3	1.8	2.002	ϕ	7.2
	% Deviation	41%	-1%	14%	-22%	n/a	n/a	n/a	-3%
	Tamimi markets-AlKharj	40	17	0.9	<1	*	*	<0.010	7.2
	Experimental value	43.5	20.5	1.3	0.0	1.3	0.005	ϕ	7.4
	% Deviation	9%	20%	48%	n/a	n/a	n/a	n/a	3%
	Tamimi markets-AlQasim	28	32	1	0.2	*	*	<0.005	7.8
	Experimental value	29.2	33.5	1.3	0.1	1.4	0.002	ϕ	7.4
	% Deviation	4%	5%	30%	n/a	n/a	n/a	n/a	-5%

		Concentrations in ppm							
	**Brand name**	**SO_4^{2}**	**Cl^-**	**F^-**	**NO_3^-**	**NO_2^-**	**Br^-**	**BrO_3^-**	**pH**
**Guidelines**	**WHO limits**	**250**	**250**	**1.5**	**50**	**3**	*****	**0.01**	**6.5-8.5**
	**SASO limits**	**150**	**150**	**1.5**	**50**	*****	*****	**0.01**	**6.5-8.5**
**Mineral**	Acqua Panna	22	7.8	<0.1	2.9	*	*	*	8
	Experimental value	19.8	8.4	0.0	2.7	3.6	ϕ	ϕ	8.2
	% Deviation	-10%	8%	n/a	-6%	n/a	n/a	n/a	2%
	Evian glass	14	10	0.06	3.8	*	*	*	7.2
	Experimental value	12.7	9.7	0.0	2.9	8.4	ϕ	ϕ	7.5
	% Deviation	-9%	-3%	n/a	-23%	n/a	n/a	n/a	4%
	Evian plastic	14	*	0.06	3.8	*	*	*	7.2
	Experimental value	12.3	10.4	0.0	3.2	8.8	0.011	ϕ	7.8
	% Deviation	-12%	n/a	n/a	-16%	n/a	n/a	n/a	8%
	Highland Spring	5.3	6.1	*	3.1	*	*	*	7.8
	Experimental value	4.8	11.6	0.0	1.9	5.9	0.018	ϕ	8.2
	% Deviation	-10%	90%	n/a	-38%	n/a	n/a	n/a	5%
	Still Scottish	<28	<11	*	<6	*	*	*	7.4
	Experimental value	8.2	6.0	0.1	2.6	4.1	ϕ	ϕ	8
	% Deviation	0%	0%	n/a	0%	n/a	n/a	n/a	8%
	Tannourine	4	10	<0.1	0.5	*	*	*	7.9
	Experimental value	13.5	6.2	0.0	0.6	5.9	0.004	ϕ	8.2
	% Deviation	237%	-38%	n/a	30%	n/a	n/a	n/a	4%

	Brand name	SO_4^{2}	Cl^-	F^-	NO_3^-	NO_2^-	Br^-	BrO_3^-	pH
		**Concentrations in ppm**							
**Guidelines**	**WHO limits**	**250**	**250**	**1.5**	**50**	**3**	*****	**0.01**	**6.5-8.5**
	**SASO limits**	**150**	**150**	**1.5**	**50**	*****	*****	**0.01**	**6.5-8.5**
**Mineral**	Volvic	9	15	*	7.3	*	*	*	7
	Experimental value	7.7	16.4	0.2	6.2	3.2	0.004	ϕ	7.5
	% Deviation	-14%	9%	n/a	-16%	n/a	n/a	n/a	7%
	Wildalp	*	2.6	*	*	0.01	*	*	7.9
	Experimental value	11.2	3.2	0.0	3.3	5.1	0.009	ϕ	7.9
	% Deviation	n/a	23%	n/a	n/a	50839 %	n/a	n/a	0%
**Artesian**	Fiji	1	9	0	1	*	*	*	7.7
	Experimental value	1.1	11.3	0.2	0.9	5.1	0.036	ϕ	7.7
	% Deviation	9%	25%	n/a	-15%	n/a	n/a	n/a	0%
	Voss glass	2.1	5.5	0.13	*	*	*	*	6.6
	Experimental value	2.5	5.2	0.1	0.2	1.0	ϕ	ϕ	6.3
	% Deviation	17%	-5%	-47%	n/a	n/a	0.015	0.015	-5%
	Voss plastic	2.1	5.5	0.13	*	*	*	*	6.6
	Experimental value	2.3	4.4	0.1	0.1	0.6	0.026	ϕ	6.3
	% Deviation	7%	-20%	-31%	n/a	n/a	n/a	n/a	-5%

	Brand name	Concentrations in ppm							
		SO_4^{2}	Cl^-	F^-	NO_3^-	NO_2^-	Br^-	BrO_3^-	pH
**Guidelines**	**WHO limits**	**250**	**250**	**1.5**	**50**	**3**	*****	**0.01**	**6.5-8.5**
	**SASO limits**	**150**	**150**	**1.5**	**50**	*****	*****	**0.01**	**6.5-8.5**
**Sparkling**	Badoit	35	54	1.2	*	*	*	*	6
	Experimental value	29.5	48.9	1.4	8.2	29.8	ϕ	ϕ	5.8
	% Deviation	-16%	-9%	18%	n/a	n/a	n/a	n/a	-3%
	Berain sparkling	9	37	1.1	0.3	*	*	<0.005	5
	Experimental value	11.9	35.4	1.3	0.5	24.2	ϕ	ϕ	4.5
	% Deviation	32%	-4%	17%	81%	n/a	n/a	n/a	-10%
	Perrier	25.3	19.5	<0.2	7.3	*	*	*	5.5
	Experimental value	23.4	19.9	0.1	8.2	25.9	0.016	ϕ	5.3
	% Deviation	-7%	2%	0%	12%	n/a	n/a	n/a	-4%
	S.pellegrino	401	49.6	0.5	*	*	*	*	5.2
	Experimental value	353.9	46.3	0.7	2.2	19.2	0.015	ϕ	5.3
	% Deviation	-12%	-7%	33%	n/a	n/a	n/a	n/a	2%
	Voss sparkling	2.1	5.5	0.13	*	*	*	*	5.2
	Experimental value	1.5	4.1	0.1	0.1	34.2	ϕ	ϕ	5.4
	% Deviation	-27%	-25%	-29%	n/a	n/a	n/a	n/a	4%

	Brand name	Concentrations in ppm							
		**SO_4^{2}**	**Cl^-**	**F^-**	**NO_3^-**	**NO_2^-**	**Br^-**	**BrO_3^-**	**pH**
**Guidelines**	**WHO limits**	**250**	**250**	**1.5**	**50**	**3**	*****	**0.01**	**6.5-8.5**
	**SASO limits**	**150**	**150**	**1.5**	**50**	*****	*****	**0.01**	**6.5-8.5**
**Tap**	Tap water 1	1.3	50.2	0	0.2	0	0.185	ϕ	ϕ
	Tap water 2	1.4	47.7	0	0.2	0	0.239	ϕ	ϕ
	Tap water 3	1.3	49.8	0	0.2	0	0.199	ϕ	ϕ
	Tap water 4	1.3	49.8	0	0.2	0	0.269	ϕ	ϕ
	Tap water 5	1.3	50.1	0	0.2	0	0.334	ϕ	ϕ
	Tap water 6	1.3	50.1	0	0.2	0	0.291	ϕ	ϕ

* Denotes values that were not provided on the composition labels of bottled waters
ϕ Denotes values that were not measured
n/a’ indicates 'not applicable' in the % deviation rows when the experimental value is below the limit of detection or when there are no label values for comparison

Table S6: Concentrations of cations and trace metals in bottled water samples measured through Inductively coupled plasma-optical emission spectroscopy in comparison to label values and to international and local standards: the World Health Organization (WHO, 2022) and the Saudi Arabian Standards Organization (SASO) (Ghrefat, 2013, Al-Omran et al., 2015). Percent deviation from label values is noted.

		Concentrations in mg/L (ppm)									
	**Brand name**	**Ca**	**Na**	**Mg**	**K**	**Fe**	**Al**	**Cu**	**Ni**	**Pb**	**Zn**
	**WHO limits**	*****	**200**	*****	*****	**0.3**	**0.9**	**2**	**0.07**	**0.01**	*****
	**SASO limits**	**200**	**100**	**150**	*****	**0.3**	**1**	**1**	*****	**0.05**	**5**
**Purified**	Aquafina	<1.0	4	20	<1.0	0.02	*	*	*	*	*
	Experimental value	0	1.25	24.06	1.16	0.00 u	0.00 u	0.00 u	0	0.00 u	0.00 u
	% Difference	0%	-69%	20%	16%	n/a	n/a	n/a	n/a	n/a	n/a
	Arwa	<1	3	21.1	<1	*	*	*	*	*	*
	Experimental value	0.03	1.53	20.38	4.65	0	0.00 u	0	0	0.00 u	0.00 u
	% Difference	0%	-49%	-3%	365%	n/a	n/a	n/a	n/a	n/a	n/a
	Berain glass	22	17	3	5	0.01	*	*	*	*	*
	Experimental value	20.28	20.06	2.55	4.23	0.00 u	0.09	0	0.00 u	0.01	0.00 u
	% Difference	-8%	18%	-15%	-15%	n/a	n/a	n/a	n/a	n/a	n/a
	Berain plastic	22	17	3	5	0.01	*	*	*	*	*
	Experimental value	22.61	14.19	3.28	6.25	0.00 u	0.00 u	0	0.00 u	0.00 u	0.01
	% Difference	3%	-17%	9%	25%	n/a	n/a	n/a	n/a	n/a	n/a
	Hana water	21	<5	9	8	*	*	*	*	*	*
	Experimental value	18.66	2.81	8.1	7.19	0.00 u	0.00 u	0	0.00 u	0.00 u	0.00 u
	% Difference	-11%	0%	-10%	-10%	n/a	n/a	n/a	n/a	n/a	n/a

	Brand name	Ca	Na	Mg	K	Fe	Al	Cu	Ni	Pb	Zn
		Concentrations in mg/L (ppm)									
	**WHO limits**	*****	**200**	*****	*****	**0.3**	**0.9**	**2**	**0.07**	**0.01**	*****
	**SASO limits**	**200**	**100**	**150**	*****	**0.3**	**1**	**1**	*****	**0.05**	**5**
**Purified**	Nestle pure life Dammam	27	9.5	2.3	0.2	<0.02	*	*	*	*	*
	Experimental value	30	8.4	3.29	0.07	0.00 u	0.00 u	0	0.00 u	0.01 u	0.00 u
	% Difference	11%	-12%	43%	-65%	n/a	n/a	n/a	n/a	n/a	n/a
	Nestle pure life Madinah	27	9.5	2.3	0.2	<0.02	*	*	*	*	*
	Experimental value	29.18	8.17	1.86	0.08	0.00 u	0.01	0.00 u	0.00 u	0.00 u	0.00 u
	% Difference	8%	-14%	-19%	-60%	n/a	n/a	n/a	n/a	n/a	n/a
	Nova	11	17	3.4	1.2	*	*	*	*	*	*
	Experimental value	17.23	17.71	5.82	1.91	0.00 u	0.01 u	0.00 u	0.00 u	0.00 u	0.00 u
	% Difference	57%	4%	71%	59%	n/a	n/a	n/a	n/a	n/a	n/a
	Tamimi markets AlKharj	10	10	10	<1	<0.1	*	*	*	*	*
	Experimental value	11.75	9.08	12.98	0.17	0.00 u	0.00 u	0.00 u	0.00 u	0.00 u	0.01 u
	% Difference	18%	-9%	30%	0%	n/a	n/a	n/a	n/a	n/a	n/a
	Tamimi markets AlQasim	21	<5	9	8	*	*	*	*	*	*
	Experimental value	18.92	3.11	8.63	7.12	0.00 u	0.00 u	0.00 u	0.00 u	0.00 u	0.00 u
	% Difference	-10%	0%	-4%	-11%	n/a	n/a	n/a	n/a	n/a	n/a

		Concentrations in mg/L (ppm)									
	**Brand name**	**Ca**	**Na**	**Mg**	**K**	**Fe**	**Al**	**Cu**	**Ni**	**Pb**	**Zn**
	**WHO limits**	*****	**200**	*****	*****	**0.3**	**0.9**	**2**	**0.07**	**0.01**	*****
	**SASO limits**	**200**	**100**	**150**	*****	**0.3**	**1**	**1**	*****	**0.05**	**5**
**Mineral**	Acqua Panna	32.2	6.5	6.6	0.8	*	*	*	*	*	*
	Experimental value	33.79	6.56	7.22	1.23	0.00 u	0.01	0.00 u	0.00 u	0.01	0.01 u
	% Difference	5%	1%	9%	54%	n/a	n/a	n/a	n/a	n/a	n/a
	Evian glass	80	6.5	26	1	*	*	*	*	*	*
	Experimental value	88	7.67	28.09	1.59	0.00 u	0.01	0.00 u	0.00 u	0	0
	% Difference	10%	18%	8%	59%	n/a	n/a	n/a	n/a	n/a	n/a
	Evian plastic	80	6.5	26	1	*	*	*	*	*	*
	Experimental value	77.88	7.23	28.45	1.56	0.00 u	0.00 u	0.00 u	0.00 u	0.00 u	0.00 u
	% Difference	-3%	11%	9%	56%	n/a	n/a	n/a	n/a	n/a	n/a
	Highland Springs	40.5	5.6	10.1	0.7	*	*	*	*	*	*
	Experimental value	38.54	7.87	21.58	1.34	0.00 u	0.00 u	0	0.00 u	0.00 u	0.01
	% Difference	-5%	41%	114%	91%	n/a	n/a	n/a	n/a	n/a	n/a
	Still Scottish	<55	<15	<16	<2	*	*	*	*	*	*
	Experimental value	28.9	10.14	10.28	1.34	0.00 u	-0.01 u	0.00 u	0.00 u	0.00 u	0.01
	% Difference	0%	0%	0%	0%	n/a	n/a	n/a	n/a	n/a	n/a
	Tannourine	50	4	13	1	<0.1	*	*	*	*	*
	Experimental value	57.88	4.18	17.13	0.91	0.00 u	0.00 u	0	0.00 u	0.00 u	0.00 u
	% Difference	16%	4%	32%	-9%	n/a	n/a	n/a	n/a	n/a	n/a
	Volvic	12	12	8	6	*	*	*	*	*	*
	Experimental value	13.33	12.22	9.24	7.1	0.00 u	0.00 u	0	0.00 u	0.00 u	0.00 u
	% Difference	11%	2%	16%	18%	n/a	n/a	n/a	n/a	n/a	n/a
	Wildalp	45	2	13	1	*	*	*	*	*	*
	Experimental value	39.78	1.83	14.79	0.33	0.00 u	-0.01 u	0.00 u	0.00 u	0.00 u	0.01
	% Difference	-12%	-9%	14%	-67%	n/a	n/a	n/a	n/a	n/a	n/a

		Concentrations in mg/L (ppm)									
	**Brand name**	**Ca**	**Na**	**Mg**	**K**	**Fe**	**Al**	**Cu**	**Ni**	**Pb**	**Zn**
	**WHO limits**	*****	**200**	*****	*****	**0.3**	**0.9**	**2**	**0.07**	**0.01**	*****
	**SASO limits**	**200**	**100**	**150**	*****	**0.3**	**1**	**1**	*****	**0.05**	**5**
**Artesian**	Fiji	18	18	15	5	*	*	*	*	*	*
	Experimental value	19.44	18.31	15.29	6.25	0.00 u	0.00 u	0	0.00 u	0.00 u	0.00 u
	% Difference	8%	2%	2%	25%	n/a	n/a	n/a	n/a	n/a	n/a
	Voss glass	3.7	3.8	0.9	*	*	*	*	*	*	*
	Experimental value	3.24	3.79	0.69	0.52	0.00 u	0.01	0	0.00 u	0.00 u	0.00 u
	% Difference	-12%	0%	-23%	n/a	n/a	n/a	n/a	n/a	n/a	n/a
	Voss plastic	3.7	3.8	0.9	*	*	*	*	*	*	*
	Experimental value	2.73	2.07	0.6	0.47	0.00 u	0.00 u	0.00 u	0.00 u	0.00 u	0.00 u
	% Difference	-26%	-46%	-33%	n/a	n/a	n/a	n/a	n/a	n/a	n/a
**Sparkling**	Badoit	153	180	80	11	*	*	*	*	*	*
	Experimental value	108.68	186.19	91.36	16	0.00 u	0.01	0.00 u	0.00 u	-0.01 u	0.00 u
	% Difference	-29%	3%	14%	45%	n/a	n/a	n/a	n/a	n/a	n/a
	Berain sparkling	20	18	2.3	0.9	0.01	*	*	*	*	*
	Experimental value	20.46	14.5	2.12	3.53	0.00 u	0	0	0.00 u	0.01	0.00 u
	% Difference	2%	-19%	-8%	292%	n/a	n/a	n/a	n/a	n/a	n/a
	Perrier	150	9.6	3.9	<1	*	*	*	*	*	*
	Experimental value	170.71	11.39	4.01	0.67	0.00 u	0	0	0.00 u	0.00 u	0.00 u
	% Difference	14%	19%	3%	0%	n/a	n/a	n/a	n/a	n/a	n/a
	S.pellegrino	166	30	49.5	2.1	*	*	*	*	*	*
	Experimental value	176.33	36.45	53.41	3.89	0.00 u	0.01	0	0.00 u	0.00 u	0.01
	% Difference	6%	22%	8%	85%	n/a	n/a	n/a	n/a	n/a	n/a

		Concentrations in mg/L (ppm)									
	**Brand name**	**Ca**	**Na**	**Mg**	**K**	**Fe**	**Al**	**Cu**	**Ni**	**Pb**	**Zn**
	**WHO limits**	*	**200**	*	*	**0.3**	**0.9**	**2**	**0.07**	**0.01**	*
	**SASO limits**	**200**	**100**	**150**	*	**0.3**	**1**	**1**	*	**0.05**	**5**
**Sparkling**	Voss sparkling	3.7	122	0.9	*	*	*	*	*	*	*
	Experimental value	3.11	119.61	0.65	0.96	0.00 u	0.01 u	0.00 u	0.00 u	0.00 u	0.00 u
	% Difference	-16%	-2%	-28%	n/a	n/a	n/a	n/a	n/a	n/a	n/a
**Tap**	Tap water 1	12.02	32.26	0.6	1.89	0.01	0.00 u	0.00 u	0.00 u	0.00 u	0.04
	Tap water 2	15.92	30.98	0.64	1.82	0.01	0.01	0.00 u	0.00 u	0.00 u	0.00 u
	Tap water 3	10.15	32.54	1.51	1.89	0.00 u	0.01	0.00 u	0.00 u	0.00 u	0.01
	Tap water 4	12.19	32.67	0.61	1.91	0.01	0.01	0.00 u	0.00 u	0.00 u	0.1
	Tap water 5	11.66	33.52	0.88	1.94	0.00 u	0.00 u	0.00 u	0.00 u	0.00 u	0.00 u
	Tap water 6	12.11	33.22	0.65	1.93	0.00 u	0.01	0.00 u	0.00 u	0.00 u	0.01

* Denotes values that were not provided on the composition labels of bottled waters
u Denotes values that were under the detection limit
n/a' indicates 'not applicable' in the % deviation rows when the experimental value is below the limit of detection or when there are no label values for comparison

2 Supplementary Methods

Materials and methods for bacterial community analysis targeting 16S V3-4 rRNA

The following information was duplicated as it was provided through supplementary documents supplied by DNASense (Aalborg, Denmark) on 29-03-2021.

1. Library preparation

Bacteria 16S rRNA gene region V3-4 sequencing libraries were prepared by a custom protocol based on an Illumina protocol (Illumina, 2015). Up to 10 ng of extracted DNA was used as template for PCR amplification of the Bacteria 16S rRNA gene region V3-4 amplicons. Each PCR reaction (25 μL) contained (12.5 μL) PCRBIO Ultra mix (PCR Biosystems, USA) and 400 nM of each forward and reverse tailed primer mix. PCR was conducted with the following program: Initial denaturation at 95 °C for 2 min, 30 cycles of amplification (95 °C for 15 s, 55 °C for 15 s, 72 °C for 50 s) and a final elongation at 72 °C for 5 min. Duplicate PCR reactions were performed for each sample and the duplicates were pooled after PCR. The forward and reverse tailed primers were designed according to (Illumina, 2015) and contain primers targeting the Bacteria 16S rRNA gene region V3-4: [341F] CCTACGGGNGGCWGCAG and [805R]

GACTACHVGGGTATCTAATCC (Herlemann et al., 2011). The primer tails enable attachment of Illumina Nextera adaptors necessary for sequencing in a subsequent PCR. The resulting amplicon libraries were purified using the standard protocol for Agencourt Ampure XP Beads (Beckman Coulter, USA) with a bead to sample ratio of 4:5. DNA was eluted in 25 μL of nuclease free water (Qiagen, Germany). DNA concentration was measured using Qubit dsDNA HS Assay kit (Thermo Fisher Scientific,USA). Gel electrophoresis using Tapestation 2200 and D1000/High sensitivity D1000 screentapes (Agilent, USA) was used to validate product size and purity of a subset of sequencing libraries. Sequencing libraries were prepared from the purified amplicon libraries using a second PCR. Each PCR reaction (25 μL) contained PCRBIO HiFi buffer (1x), PCRBIO HiFi Polymerase (1 U/reaction) (PCRBiosystems, UK), adaptor mix (400 nM of each forward and reverse) and up to 10 ng of amplicon library template. PCR was conducted with the following program: Initial denaturation at 95 °C for 2 min, 8 cycles of amplification (95°C for 20 s, 55 °C for 30 s, 72 °C for 60 s) and a final elongation at 72°C for 5 min. The resulting sequencing libraries were purified using the standard protocol for Agencourt Ampure XP Beads (Beckman Coulter, USA) with a bead to sample ratio of 4:5. DNA was eluted in 25 μL of nuclease free water (Qiagen, Germany). DNA concentration was measured using Qubit dsDNA HS Assay kit (Thermo Fisher Scientific, USA). Gel electrophoresis using Tapestation 2200 and D1000/High sensitivity D1000 screentapes (Agilent, USA) was used to validate product size and purity of a subset of sequencing libraries.

2. DNA sequencing

The purified sequencing libraries were pooled in equimolar concentrations and diluted to 2 nM. The samples were paired-end sequenced (2x300 bp) on a MiSeq (Illumina, USA) using a MiSeq Reagent kit v3 (Illumina, USA) following the standard guidelines for preparing and loading amples on the MiSeq. >10% PhiX control library was spiked in to overcome low complexity issues often observed with amplicon samples.

References

AL-OMRAN, A., AL-BARAKAH, F., ALTUQUQ, A., ALY, A. & NADEEM, M. 2015. Drinking water quality assessment and water quality index of Riyadh, Saudi Arabia. *Water Quality Research Journal of Canada,* 50**,** 287-296.

BUTTON, D., ROBERTSON, B. R. J. A. & MICROBIOLOGY, E. 2001. Determination of DNA content of aquatic bacteria by flow cytometry. 67**,** 1636-1645.
GHREFAT, H. 2013. Classification and evaluation of commercial bottled drinking waters in Saudi Arabia. *Research Journal of Environmental and Earth Sciences*, 5**,** 210-218.
HAMMES, F., BERNEY, M., WANG, Y., VITAL, M., KÖSTER, O. & EGLI, T. 2008. Flow-cytometric total bacterial cell counts as a descriptive microbiological parameter for drinking water treatment processes. *Water Research,* 42**,** 269-277.
HERLEMANN, D. P., LABRENZ, M., JÜRGENS, K., BERTILSSON, S., WANIEK, J. J. & ANDERSSON, A. F. 2011. Transitions in bacterial communities along the 2000 km salinity gradient of the Baltic Sea. *The ISME journal,* 5**,** 1571-1579.
ILLUMINA, I. 2015. 16S metagenomic sequencing library preparation, part# 15044223 Rev. 1213**,** 1214.
WHO 2022. *Guidelines for drinking-water quality: incorporating the first and second addenda*, World Health Organization.
